# Single-Cell Proteomics and Tumor RNAseq Identify Novel Pathways Associated With Clofazimine Sensitivity in PI- and IMiD- Resistant Myeloma, and Putative Stem-Like Cells

**DOI:** 10.3389/fonc.2022.842200

**Published:** 2022-05-11

**Authors:** Harish Kumar, Suman Mazumder, Neeraj Sharma, Sayak Chakravarti, Mark D. Long, Nathalie Meurice, Joachim Petit, Song Liu, Marta Chesi, Sabyasachi Sanyal, A. Keith Stewart, Shaji Kumar, Leif Bergsagel, S. Vincent Rajkumar, Linda B. Baughn, Brian G. Van Ness, Amit Kumar Mitra

**Affiliations:** ^1^ Department of Drug Discovery and Development, Harrison College of Pharmacy, Auburn University, Auburn, AL, United States; ^2^ Center for Pharmacogenomics and Single-Cell Omics (AUPharmGx), Harrison College of Pharmacy, Auburn University, Auburn, AL, United States; ^3^ Division of Laboratory Genetics, Department of Laboratory Medicine and Pathology, Mayo Clinic, Rochester, MN, United States; ^4^ Department of Biostatistics and Bioinformatics, Roswell Park Comprehensive Cancer Center, Buffalo, NY, United States; ^5^ Division of Hematology/Oncology, Mayo Clinic Arizona, Scottsdale, AZ, United States; ^6^ Biochemistry Division, CSIR-Central Drug Research Institute, Lucknow, India; ^7^ Division of Hematology, Department of Internal Medicine, Mayo Clinic, Rochester, MN, United States; ^8^ Department of Genetics, Cell Biology and Development, University of Minnesota, Minneapolis, MN, United States

**Keywords:** CyTOF, RNAseq, myeloma, clofazimine, proteasome inhibitors (PI), immunomodulatory drugs (IMiDs), side population (SP), human myeloma cell lines (HMCLs)

## Abstract

Multiple myeloma (MM) is an incurable plasma cell malignancy with dose-limiting toxicities and inter-individual variation in response/resistance to the standard-of-care/primary drugs, proteasome inhibitors (PIs), and immunomodulatory derivatives (IMiDs). Although newer therapeutic options are potentially highly efficacious, their costs outweigh the effectiveness. Previously, we have established that clofazimine (CLF) activates peroxisome proliferator-activated receptor-γ, synergizes with primary therapies, and targets cancer stem-like cells (CSCs) in drug-resistant chronic myeloid leukemia (CML) patients. In this study, we used a panel of human myeloma cell lines as *in vitro* model systems representing drug-sensitive, innate/refractory, and clonally-derived acquired/relapsed PI- and cereblon (CRBN)-negative IMiD-resistant myeloma and bone marrow-derived CD138+ primary myeloma cells obtained from patients as *ex vivo* models to demonstrate that CLF shows significant cytotoxicity against drug-resistant myeloma as single-agent and in combination with PIs and IMiDs. Next, using genome-wide transcriptome analysis (RNA-sequencing), single-cell proteomics (CyTOF; Cytometry by time-of-flight), and ingenuity pathway analysis (IPA), we identified novel pathways associated with CLF efficacy, including induction of ER stress, autophagy, mitochondrial dysfunction, oxidative phosphorylation, enhancement of downstream cascade of p65-NFkB-IRF4-Myc downregulation, and ROS-dependent apoptotic cell death in myeloma. Further, we also showed that CLF is effective in killing rare refractory subclones like side populations that have been referred to as myeloma stem-like cells. Since CLF is an FDA-approved drug and also on WHO’s list of safe and effective essential medicines, it has strong potential to be rapidly re-purposed as a safe and cost-effective anti-myeloma drug.

## Introduction

Multiple myeloma (MM) is an incurable neoplasm characterized by clonal expansion of malignant antibody-producing post-germinal- center B-cell-derived plasma cells within the bone marrow (1). Myeloma is the second-most common hematopoietic malignancy in the United States, with an estimated 34,920 new cases and 12,410 deaths in 2021 (American Cancer Society; https://www.cancer.org/cancer/multiple-myeloma/about/key-statistics.html). Proteasome inhibitors (PIs; bortezomib/Bz/Velcade, carfilzomib/Cz, and Ixazomib/MLN9708/Ix) are standard-of-care/primary chemotherapeutic agents for relapsed and refractory myeloma that impede tumor metastasis and angiogenesis by accelerating unfolded protein response (UPR) and by interfering with the NF-κB-enabled regulation of cell adhesion-mediated drug resistance (1–5). Combination therapy regimens incorporating PIs and immunomodulatory drugs (IMiDs; Lenalidomide/Revlimid, Pomalidomide) as backbone have significantly improved treatment responses, including progression-free survival (PFS) and overall survival (OS) (6, 7). However, despite these and other recent improvements in therapies, myeloma still remains a difficult-to-cure disease with dose-limiting toxicities and drug resistance and a median survival rate of only around 7 years (3, 4, 8). Moreover, a recent study on the cost-effectiveness of anti-myeloma drugs suggested that although the current therapeutic regimens including novel treatments (like monoclonal antibodies and Chimeric antigen receptor or CAR-T-cell therapy) are highly promising, the costs outweigh the effectiveness based on willingness-to-pay thresholds (9). *Therefore, our goal was to search for new secondary therapeutic options with lower costs and higher cost-effectiveness to treat drug resistance in myeloma.*

Previously, we have demonstrated that Clofazimine (CLF), an anti-leprosy drug, activates peroxisome proliferator-activated receptor-γ and synergizes with the standard-of-care drug imatinib for the treatment of chronic myeloid leukemia (CML) (10). Although two studies have shown the efficacy of CLF treatment in multiple myeloma, none of these have explored the synergistic effect of CLF in combination with PI or IMiD therapy or the impact of CLF-based therapy using model systems representing the wide inter- and intra- tumor heterogeneity in myeloma drug response (11, 12). Further, the potential mechanisms underpinning CLF as an anti-myeloma drug have not been understood fully so far. Therefore, in this study, we used a diverse panel of human myeloma cell lines and patient-derived primary myeloma cells to investigate the potential of CLF as an anti-myeloma drug against inter-tumor and intra-tumor heterogeneity in PI and IMiD-resistant myeloma. Furthermore, using genome-wide transcriptomics (tumor mRNA-sequencing) and single-cell proteomics (CyTOF or Cytometry by time-of-flight), we also identified several genes and potential molecular networks involved in the CLF mechanism of action and drug synergy with PIs and IMiDs.

Drug resistance is a manifestation of significant complexity and heterogeneity at the molecular level (4, 13, 14). In addition, the presence of rare subpopulations of tumor cells with stem cell-like properties like greater clonogenicity, self-renewal, and differentiation capacities, are believed to significantly contribute towards treatment-refractory phenotypes in various cancers, including myeloma (15). Since our previous study had shown that CLF erodes quiescent stem-cell populations (CD34+CD38-, CFSE-bright) in drug-resistant CML patients (10), we also showed that CLF kills quiescent/dormant cells, ALDH+ cells, and side populations (SPs), collectively referred to as putative stem-like cells in myeloma, with treatment-refractory phenotypes (16–19). *We propose clinical efficacy studies in relapsed/refractory myeloma using clofazimine-based drug combination regimens.*

## Materials and Methods

### Human Myeloma Cell Lines (HMCLs)

We have compiled a large panel of HMCLs representing *innate sensitivity and resistance to PIs and IMiDs (similar to refractory disease)*, as well as >10 pairs of parental and clonally-derived PI/IMiD-resistant HMCLs (P vs.R pairs; generated using dose escalation over a period of time) representing *acquired or emerging drug resistance*, and encompassing the broad spectrum of biological and genetic heterogeneity of myeloma (20). The human myeloma cell lines (HMCLs) were obtained from Dr. Michael Kuehl (NIH), Dr. Leif Bergsagel (Mayo Clinic, Scottsdale, AZ), and Dr. Jonathan Keats (The Translational Genomics Research Institute (TGen) Phoenix, AZ) as described in our earlier publication (20). The clonally related acquired PI-resistant HMCLs were generated from parental cell lines by dose escalation of bortezomib using pulses of once-weekly bortezomib treatment as described previously (21). These HMCLs were used as *in vitro* model systems to screen and re-purpose drugs for the management of PI-resistant myeloma.

We also found that *in vitro* drug responses of the four PIs (Bz, Cz, Opz, and Ix) were highly correlated in HMCLs (20). Therefore, we used Ixazomib, a second-generation PI, as a representative PI for our studies.

The lenalidomide (IMiD)-resistant cell line, MM1SLenR, was obtained as a gift from Dr. Keith Stewart, Mayo Clinic. The cell lines were authenticated at source and tested randomly at regular intervals for mycoplasma negativity. HMCLs were maintained in HMCL media supplemented with IL-6 (22).

### Patient Primary Myeloma Cells (PMCs)

Bone marrow-derived CD138+ primary myeloma cells were obtained from patients (n=16) following written informed consent at Mayo Clinic with prior IRB approval from the Mayo Clinic review board and used as *ex vivo* model systems. Written informed consent was received from participants prior to inclusion in the study. Participants have been identified by number, not by name. The primary samples were cultured as total bone marrow and put in culture to retain the patients’ natural BM microenvironment. Cells of interest were CD138+-gated during analysis.

### Drugs and Reagents

Drugs, reagents, antibodies, and kits are listed in **Supplementary Table S1**.

### *In Vitro* Chemosensitivity Assays and Drug Synergy Analysis

HMCLs were treated with increasing concentrations of CLF, PIs (represented by Ix), and IMiDs (represented by Len) as single agents or in combination for 48h, and cytotoxicity assays were performed using CellTiter-Glo^®^ Luminescent cell viability assay (Promega Madison, WI). Luminescence was recorded in a Neo2 Microplate Reader (Biotek), and half-maximal inhibitory concentration (IC_50_) values were determined using by calculating the nonlinear regression using sigmoidal dose-response equation (variable slope). The drug cytotoxicity data and IC_50_ values were used to compare response and to determine synergy by Calcusyn software (Biosoft; Chou-Talalay’s CI theorem) (23). CI value<0.9 denotes synergism.

### Apoptosis Assays

Cells were cultured for 48h in the presence of indicated concentrations of compounds, harvested and washed, and incubated with Annexin V-FITC (BD Biosciences) and Propidium Iodide for 15min at room temperature in the dark. Apoptosis was measured by BD LSR II flow cytometry (BD Biosciences).

Caspase-3/7 activity assay was performed on the HMCLs using Caspase-Glo 3/7 luminescent assay kit according to manufacturer’s instructions (Promega Madison, WI). Briefly, 1×10^6^/ml cells were cultured in 6 well plates and treated with either CLF alone and/or combination for indicated times. After harvesting, cells were washed twice with cold PBS and resuspended with Caspase-Glo 3/7 reagents. Reactions were incubated for 1h at 37°C, and luminescence was determined using Synergy 2 Microplate Reader (Biotek). Cell death by apoptosis was also measured by immunoblotting analysis.

### Cell Cycle Analysis

HMCLs were seeded in 6-well plates and incubated for 48h following treatment with 10µM CLF. Cells were then harvested, washed, and incubated with Propidium Iodide for 30min at room temperature. Cell cycle progression was measured using a BD LSR II flow cytometer (BD Biosciences). Flow cytometry data analysis was performed using FlowJo software (BD Biosciences).

### Colony-Forming Cells (CFCs) Assay

Colony formation potential of untreated and drug-treated HMCLs was accessed using Methylcellulose-based assay according to manufacturer’s instructions (Methocult, Stem Cell Technologies). Briefly, myeloma cells were treated with either CLF alone and/or combination with PI for the indicated time period. Following incubation, cells were harvested, washed with PBS, and methylcellulose-based media (Methocult) was added to cells pellets, and colonies were allowed to grow for 4 weeks in 6-well plates. Images were captured after 4 weeks with an inverted microscope (Olympus, 4x/20x lens with color camera), and cell colony numbers were counted by Image J software (NIH).

### Aldeflour Activity Assay

Aldehyde dehydrogenase (ALDH) activity was assessed using the Aldefluor assay kit according to the manufacturer’s instructions (Stem Cell Technologies). Briefly, 1×10^6^ myeloma cells were treated with CLF-based regimens for 24h, harvested, and resuspended in 1ml Aldefluor assay buffer containing the ALDH substrate BODIPY-aminoacetaldehyde (BAAA). Negative control samples were treated with 5μl of diethylaminobenzaldehyde (DEAB) as an inhibitor of ALDH1 enzymatic activity. Cells were incubated for 30–45 minutes at 37 °C, then washed twice, and suspended in Aldefluor assay buffer. The brightly fluorescent ALDH^+^ cells were detected by BD LSR II flowcytometry.

### Carboxyfluorescein Succinimidyl Ester (CFSE) Assay

For assessing apoptosis in quiescent CD138+ cells, myeloma cells were first stained with carboxyfluorescein succinimidyl ester (CFSE; Invitrogen) for 30 min, then washed and resuspended in RPMI1640 medium and were incubated at 37°C overnight. The next day, cells were treated and maintained further for 4 days with supplementation of the drugs every 48h. Cells were then harvested, washed, and were labeled with CD138-APC and annexin/V-FITC antibodies and then gated into non-dividing (CD138+CFSEbright) and dividing (CD138+CFSEdim) cell populations based on CFSE fluorescence intensity using a flow cytometer. Cells cultured in the presence of colchicine (100ng/ml; Sigma) were used to assess the range of CFSE fluorescence exhibited by cells that were undivided at the end of the culture time.

### Side Population Analysis

Side population cells were investigated using DyeCycle Violet (cell-permeable DNA binding) assay according to manufacturer’s instructions (Thermofisher). Briefly, 1×10^6^/ml cells were cultured in 6 well plates and treated with either CLF alone and/or combination for indicated times. After 96h, cells were stained with 10μM Vybrant DyeCycle Violet (DCV; Molecular Probes, Eugene, OR) for 90 min at 37°C. Samples incubated with 100μM verapamil (Sigma-Aldrich) for 30 min were used as positive control. Following dye incubation, cells were washed twice with ice-cold PBS, chilled on ice, and immediately analyzed by flow cytometry.

### Determination of Total Cellular Reactive Oxygen Species (ROS)

Cells were treated with CLF-based regiments for 24hr. After 24h, cells were incubated with 10μM DCFDA in RPMI (Phenol red-free) medium at 37°C for 30min, washed twice with phosphate buffer saline (PBS), and ROS production was measured using Synergy 2 Microplate Reader (Biotek).

### Measurement of Superoxide Levels

Myeloma cells treated with CLF-based regimens for 24h were incubated with 2.5 μM DHE (in RPMI) for 15 min in the dark at 37°C. Cells were then washed once with PBS, and red fluorescence was detected by flow cytometry.

### Mitochondrial Membrane Potential (MMP) Measurement

CLF-treated (24h) cells were incubated with 5μM JC-1 dye for 15 min in the dark at 37°C and washed twice in PBS, and then analyzed for red and green fluorescence by flow cytometry.

### Gene Expression Profiling (GEP) Analysis

Cells were plated at a density of 4X10^5^/ml cells and were treated with either CLF alone, Ixa alone, or combination. 24hrs after incubation, high-quality RNA was isolated using QIAshredder and RNAeasy mini kit (Qiagen). RNA concentration and integrity were determined using the Nanodrop-8000 and Agilent 2100 Bioanalyzer and stored at -80°C. RNA integrity number (RIN) threshold of 8 was used for RNA-seq analysis. RNA-seq libraries were constructed using Illumina TruSeq RNA sample preparation kit v2. Libraries were then size-selected to generate inserts of ~200bp. Next-generation RNA sequencing was performed on Illumina’s NovaSeq platform using 150bp paired-end protocol with a depth of >20million reads-per-sample.

### RNAseq Data Analysis

Gene expression data was pre-processed, filtered (genes with mean counts<10 were removed), and GEP (CPM – counts per million) data was analyzed further using Partek Flow package to perform differential expression testing to identify GEP signatures. LS (least squares) mean values were calculated for each group as the linear combination or sum of the estimated means from the linear model. LS mean is model-dependent and produces more accurate, unbiased estimate of the group means even in unbalanced data. We used an LS Mean threshold of >=1 for downstream analysis. We used Gene Specific Analysis (GSA) to perform differential gene expression analysis between groups that applies limma, an empirical Bayesian method, to detect the differentially expressed (DE) genes. The advantage of limma compared to traditional t-test is that limma provides a moderated t-test statistic by shrinking the variance statistics, therefore, improving the statistical power. Mean fold-change>|1| and p<0.05 was considered as threshold for reporting significant differential gene expression. Heatmaps were generated using unsupervised hierarchical clustering (HC) analysis based on the top DE genes (DEGs).

**Ingenuity pathway analysis (IPA)** software was used to identify, i) the most significantly affected molecular pathways, ii) upstream regulator molecules like microRNA and transcription factors, iii) downstream effects and biological processes, and iv) causal networks predicted to be activated or inhibited in response to treatment based on the most significant DE genes (24).

### Single-Cell Proteomics

Mass cytometry (CyTOF) analysis is a single-cell proteomics methodology that combines flow cytometry and elemental mass spectrometry. Thirty-seven antibody targets directed against cell surface (neoplastic myeloma) and intracellular markers (immune tumor microenvironment) were utilized for immunophenotyping panels. The antibody markers and respective metal conjugates are described in **Supplementary Table S2**. 1-3million untreated or treated (for 24h) HMCLs or primary human myeloma cells were used for CyTOF staining. Cells were stained with cisplatin (1:10,000 dilutions in RPMI1640 media without FBS) at 37°C for 5 minutes, washed with media with 10% FBS, spun at 300 x g, and incubated with media with 10% FBS for 15 min. This was followed by another wash, spin, and the pellet was resuspended in 1x Fix buffer for 10 minutes at room temperature. HMCL samples, along with lyophilized healthy human PBMC (Veri-Cells PBMC: control cells; BioLegend), were washed using Maxpar cell staining buffer (MCSB) (Fluidigm). A total of 16 µL of the surface antibody cocktail was added to each tube containing each cell suspension in 100 µL of MCSB and incubated for 30 minutes at room temperature. Cells were washed with MCSB to remove excess antibodies and stored overnight at -80 C after methanol fixation. Cells were washed with MCSB and incubated with 20 µL of intracellular antibody cocktail for 30 minutes at room temperature, followed by wash and fixation with fresh 1.6% formaldehyde solution for 10 minutes at room temperature. Cells were washed with MCSB and resuspended in 1 ml of Cell-ID™ Intercalator solution (1:4000 dilution in permeabilization buffer, Maxper Fix, and Perm Buffer). All samples were barcoded using cell-ID™ 20-Plex Barcoding Kit (Fluidigm), which were then stained, washed with MSCB, and acquired as a single multiplexed sample using Maxpar Cell Acquisition solution contained calibration beads.

Samples were loaded onto a Helios CyTOF system (Fluidigm) using an attached autosampler and were acquired at a rate of 200-400 events per second. Data were collected as.FCS files using the CyTOF Software (Version 6.7.1014, Fluidigm). After acquisition, intrafile signal drift was normalized to the acquired calibration bead signal using the CyTOF Software.

### CyTOF Data Analysis

For CyTOF data analysis, Cytobank software version 7.3.0 (Santa Clara, CA) was used for cleanup of cell debris, removal of doublets and dead cells, and analysis of cleaned fcs files. Clustering, dimensionality reduction (to 10,000 events per file), and the visualization of cell population cluster map were performed by t-SNE. Relative marker intensities and cluster abundances per sample were visualized by a heatmap.

### Western Blotting

Cells treated with CLF, PI, or IMiD as single-agent or as a combination for 24h were harvested, washed, and lysed using radioimmunoprecipitation assay (RIPA) lysis buffer containing 50mM Tris-HCl, pH 7.5, 150mM NaCl, 1% NP40, 5mM EDTA, 1mM DTT, phosphatase and protease inhibitors cocktail (Sigma), and incubated on ice for 15min. Samples were then centrifuged at 14000 rpm at 4°C for 30mins. The supernatant was then aspirated, total protein was isolated and quantified using Pierce BCA Protein Assay Kit (Thermo Scientific). Samples were solubilized in SDS-PAGE sample buffer, and equal amounts of protein were loaded per lane of 10% SDS polyacrylamide gels and transferred onto PVDF membranes (Millipore; Billerica, MA). Membranes were blocked in TBS with SuperBlock blocking buffer (Thermo Fisher). Membranes were then incubated with primary antibodies and secondary antibodies in TBS with 0.2% Tween 20 and 2.5% BSA. Immunoreactivity was detected by Chemiluminescent HRP Substrate (Bio-Rad), and the exposed image was captured using a ChemiDoc MP Imaging System (Bio-Rad). Densitometry analysis was performed (in triplicates) using Image J software.

### Quantitative Reverse Transcriptase Polymerase Chain Reaction (qRT-PCR)

Total RNA isolation from untreated HMCLs and quantification were performed using QIAshredder and RNAeasy mini kit (Qiagen). RNA concentration was determined using the Nanodrop-8000 spectrophotometer, and cDNA was prepared using QuantiTect Reverse Transcription kit (Qiagen). Following reverse transcription, TaqMan gene expression assay was performed using AHR mRNA specific TaqMan primers (TaqMan Real-Time PCR Assays) and TaqMan Fast Advanced Master Mix in CFX96 Touch Real-Time PCR Detection System (Bio-Rad, Hercules, CA). Relative AHR expression in the myeloma lines was calculated using the ΔCt method following normalization with Beta-Actin expression (housekeeping gene).

### *Ex Vivo* Direct-to-Drug Screening Assay

The drugs were screened in a 7-point, 10-fold dilution, with the highest concentration at 10 µM, and incubated for 24h. Anti-MM activity was assessed through cellular viability, evaluated with CellTiter Glo. The mid-point EC_50_, percentage of maximum inhibition was calculated using the TIBCO Spotfire^®^ v.7.0.0 software, and the area under the curve (AUC) was calculated with the GraphPad Prism v.8 software (25). This data was compared to *in vitro* response profiles and linked through integrated analyses to CyTOF profiles and multi-omics outcomes.

### Statistical Analysis

All statistical analyses were performed using R and GraphPad Prism. All tests were two-sided, and p<0.05 was considered statistically significant.

## Results

### CLF Induces Loss of Viability in HMCLs and PMCs

**Figure 1:**
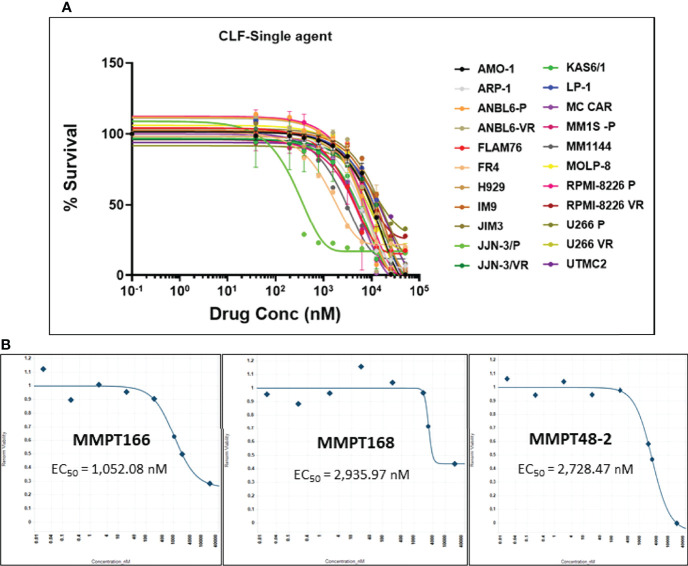
CLF decreases the *in vitro* and *ex vivo* cell viability in multiple myeloma. **(A)** Response to single-agent CLF treatment in HMCLs (human myeloma cell lines). **(B)** Representative *ex vivo* CLF dose-response plots in patient bone marrow-derived primary myeloma cells.

First, we evaluated the single-agent *in vitro* cytotoxicity of CLF for anti-myeloma activity in our HMCL panel, representing wide variation in PI and IMiD responses (IC_50_) (**Figure 1A**). We found that CLF alone showed very potent inhibition of cell viability in HMCLs representing sensitive as well as innate and clonally-derived resistant HMCLs. The single-agent IC_50_ (48h) values of CLF were between 0.2µM- 20.5µM. Next, we compared the link between CLF IC_50_ of the myeloma cell lines and MM molecular/cytogenetic abnormalities (22). Our results showed no significant association of CLF response with cytogenetic abnormalities (data not shown).

Further, bone marrow-derived CD138-positive PMCs were obtained from patients (n=12) at Mayo Clinic and used as *ex vivo* model systems. Using our established direct-to-drug screening assay, we screened each patient’s tumor in a Phase 0 assay for drug sensitivity to single-agent CLF (25). The *ex vivo* CLF EC_50_ values (1-15 µM; minimum EC_50_ 1052.1nM, maximum EC_50_ 15210nM, median EC_50_ 2408.7nM) were within the *in vitro* IC_50_ range. **Figure 1B** shows representative CLF single-agent *ex vivo* cytotoxicity plots in myeloma patients.

### CLF Shows Synergy With Proteasome Inhibitors and IMiDs

**Figure 2:**
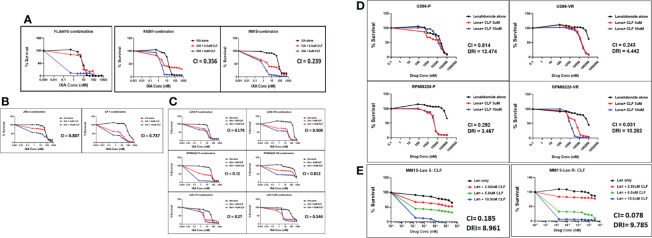
Clofazimine synergizes with Proteasome inhibitors and Immunomodulatory drugs (IMiDs) in multiple myeloma. CLF + PI (represented by Ixazomib) treatment in **(A)** Innate-sensitive myeloma cell lines; **(B)** Innate-resistant myeloma cell lines. **(C)** Parental and clonally-derived acquired resistant myeloma cell lines. CLF + IMiD (represented by Lenalidomide) treatment in **(D)** Parental and clonally-derived acquired PI-resistant myeloma pairs; and **(E)**. IMiD sensitive/resistant pair. (CI – Combination index calculated using Chou-Talalay’s CI theorem).

Next, we tested the cytotoxicity of CLF in combination with PIs (represented by Ixazomib; **Figures 2A–C**) or IMiDs (represented by Lenalidomide; **Figures 2D, E** **)** in HMCLs representing innate-sensitive (FLAM76, KAS6/1, MM1S), innate-resistance (JIM-3, LP-1), and acquired- PI or IMiD resistance (U266 P/VR, RPMI8226 P/VR, JJN-3 P/VR, and MM1S P/LenR). The CLF+PI and CLF+Len combination index (CI) values calculated using the Calcusyn program were consistently less than 0.9 (**Figure S1**), indicating synergy (26). Further, CLF improved the therapeutic index of PI and IMiD administration to the cells and decreased the amount of PI/IMiD required to achieve effective responses, as indicated by dose reduction index (DRI) values and predicted decrease in IC_50_ (nM concentration).

Although the CLF doses used in combination treatments were in the micromolar concentration range, this is within the safe dose range of 0.84-8.4µM, which corresponds to human plasma Cmax of 0.4-4mg/L (10).

### CyTOF Analysis Reveals CLF-Induced Key Proteomic Changes at Bulk and Subclonal Levels

**Figure 3:**
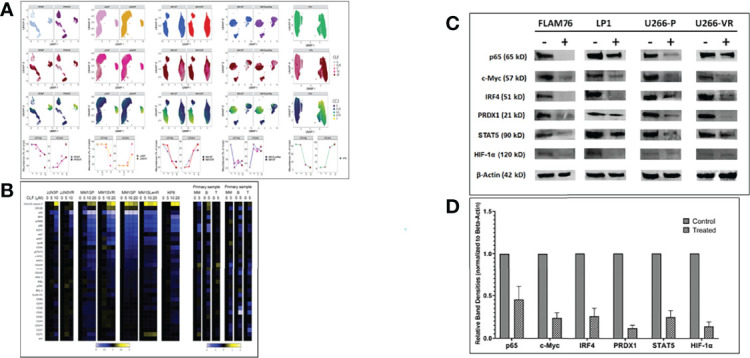
CyTOF analysis in multiple myeloma cell lines (representing sensitive, PI-resistance and IMiD-resistance) and patient primary cells. **(A)** CLF induces elevated cleaved caspase 3 levels. Samples were treated with CLF or DMSO and Gated on LIVE cells. Each ‘column’ represents a cell line pair (except for KP6, which is just the parental). The first three ‘rows’ are UMAP plots colored by cell line, CLF dose, and cc3 expression. For the final ‘row’, the FlowSOM meta-cluster results were condensed into cc3 positive and negative cell subsets based on cc3 expression UMAPs and plotted over CLF dose. cc3 is induced in all lines. **(B)** CLF-treatment results in downregulation of genes associated with myeloma cell survival. Representative heatmap for CyTOF analysis is shown for sensitive and PI-resistant, IMiD-resistant, and patient primary cells showing expression of the complete panel. Heatplot was generated in Cytobank displaying the transformed ratio normalized to the first column (DMSO control) of the median of each marker. CyTOF analysis shows shifts in a number of myeloma cell survival markers following clofazimine treatment (10uM), including IRF4, IKZF1 (Ikaros), IKZF3 (Aiolos), CD229, CD27, pS6, pERK, and IkBa. CLF acts as a PPAR-gamma agonist that synergizes with PIs to enhance the downstream cascade of p65/NFkB/IRF4/Myc downregulation followed by ROS-dependent apoptotic cell death. **(C)** Western blotting. Representative figure showing pre- vs. post-treatment (24hr) immunoblotting analysis of proteins involved in the p65/NFkB/IRF4/Myc axis and ROS-dependent apoptotic pathways. Beta-actin was used for the normalization of the Western blots. **(D)** Densitometry analysis showing relative band densities between untreated vs. treated cells lines. Band densities were compared to Beta-actin.

We performed Mass Cytometry (CyTOF) analysis to assess CLF-induced changes in phenotypic and functional markers in myeloma cells on a single-cell level and identify unique subgroups that change in relation to disease progression. CyTOF analysis was performed on 77 total samples across 7 Experiments/Batches. This included 4 isogenic sensitive/acquired PI and IMiD resistant pairs (U266, MM1S, RPMI8226, JJN3), 8 innate-sensitive cell lines, and 7 innate-resistant HMCLs. Batch correction was performed for combining samples. Similar clusters across all samples were grouped to compare sub-populations and to calculate the proportion of cells with increases or decreases in markers for each sample. CyTOF analysis revealed distinct cluster of cells defined by elevated cleaved caspase levels in all cell lines and primary samples, which was enriched for cells exposed to high dose CLF (**Figure 3A**).

Myeloma cells are addicted to several proteins like c-Myc, IRF4, and IKZF1. Our pre- vs. post-treatment differential expression analysis using CyTOF (**Figure 3B**) and immunoblotting (**Figure 3C**) revealed shifts in a majority of these markers following clofazimine treatment, including IRF4, IKZF1, IKZF3, CD229, CD27, pS6, pERK, and IkBa.

Furthermore, we had earlier found that CLF also suppresses STAT expression in CML and consequently downregulated stem cell maintenance factors like hypoxia-inducible factor-1α and -2α and Cbp/P300 interacting transactivator with Glu/Asp-rich carboxy-terminal domain 2 (CITED2) (10). Concurrently, we also showed downregulation of STAT5 and HIF-1α in myeloma following CLF treatment (**Figure 3D** **).**

### CLF Induces Apoptosis *via* Mitochondrial-Mediated Pathway in Myeloma

**Figure 4:**
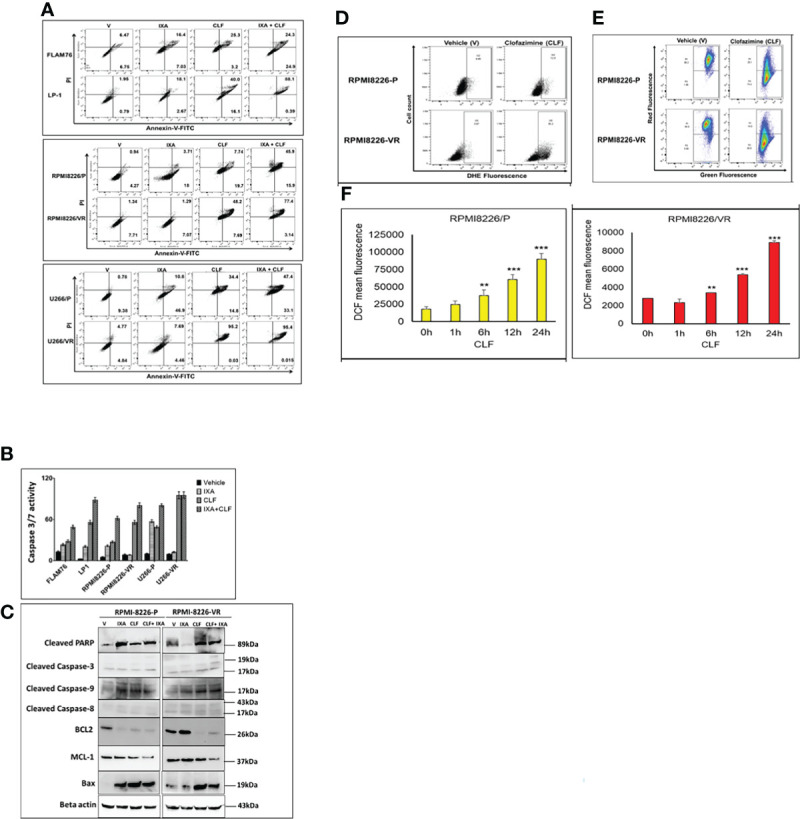
**(A–C)** CLF induces apoptosis in sensitive and innate resistant MM cell lines. **(A)** Apoptosis assays were performed on Innate and Acquired sensitive-resistant pairs by Annexin V-FITC kit (BD Biosciences) using flow-cytometry (BD LSR II) flow cytometry. **(B)** Caspase-3/7 activity assays were performed using Caspase-Glo 3/7 luminescent assay (Promega Madison, WI) using Synergy 2 Microplate Reader (Biotek). **(C)** Western blot results represent CLF treatment results in dysregulation of apoptotic proteins. Larger blots of PARP cleavage and caspase cleavage are provided in **Supplementary Figure S10**. **(D–F)** CLF induces Super-oxide levels, intra-cellular ROS generation, and mitochondrial membrane potential (MMP) in myeloma cell lines. Representative figures showing the RPMI8226 P/VR cell line pair. **(D)** Super-oxide. Cellular superoxide anions were measured by using the fluorescent dye DHE (Sigma), and red fluorescence was detected by flow cytometry. **(E)** ROS generation. DCFDA (Sigma), which shows fluorescence when it is oxidized, was used to measure intracellular ROS production by flow cytometry. **(F)** Mitochondrial membrane potential (MMP). Mitochondrial membrane potential was assessed using JC-1 (Sigma), a cationic carbocyanine dye that accumulates in mitochondria. Cells were incubated with 5μM JC-1 dye for 15 min in the dark at 37°C, washed twice in PBS, and then analyzed for red and green fluorescence by flow cytometry. *p < 0.05, **p < 0.01, ***p < 0.001.

Next, to confirm whether the loss of cell viability was indeed due to apoptosis (as indicated by CyTOF analysis results; **Figure 2**), we performed annexin V staining using flow-cytometry. CLF induced significant apoptosis either alone or in combination with 15nm Ixazomib in HMCLs (**Figure 4A**). CLF-induced caspase-3 activity was also confirmed by luminescent-based Caspase 3/7 assay (Promega) (**Figure 4B**). We observed that CLF activated caspase-3 and 9 but not 8, indicating that CLF-induced apoptosis was dependent on mitochondria-mediated pathway (Western blotting; **Figure 4C**). This is consistent with decrease in the anti-apoptotic/survival markers Bcl2, Mcl-1 and increase in Bax expression (**Figure 4C**). Furthermore, using Caspase 3/7 assay (**Figure S2A**) and western blotting (**Figure S2B**), we demonstrated that CLF treatment results in induction of apoptosis in myeloma lines. When gated for live cells, the percentage of cells in G0/G1, S, G2/M, and sub G0/G1 phases in pre- vs. post- CLF treatment HMCLs are shown in **Figure S2C**.

Previously, it has been reported that CLF imparts its anti-bacterial actions by generating reactive oxygen species (ROS), particularly superoxides and hydrogen peroxide (H_2_O_2_). DCFDA (Sigma), which shows fluorescence when it is oxidized, was used to measure intracellular ROS. Cellular superoxide anions were measured by using the fluorescent dye DHE (Sigma). Mitochondrial membrane potential was assessed using JC-1 (Sigma). JC-1 is a cationic carbocyanine dye that accumulates in mitochondria. Representative plots demonstrate that we observed induction of cellular superoxide anions (**Figure 4D**) and intracellular ROS production (**Figure 4E**) that causes mitochondrial membrane depolarization following CLF treatment in the cell pair RPMI8226P/VR (**Figure 4F**). Similar results were also obtained for the U266P/VR cell line pair (**Figures S3A, B**).

### CLF Inhibited Myeloma Colony Formation, Aldefluor Activity and Eroded Quiescent and Side Population Cells

We observed CLF alone significantly reduced colony number as well as colony size when compared to control or Ixazomib. Combination of CLF with Ixazomib further reduced the colony numbers. Importantly, CLF significantly reduced colony formation in PI-resistant lines (RPMI826 P/VR and U266 P/VR) (**Figure S4**).

**Figure 5:**
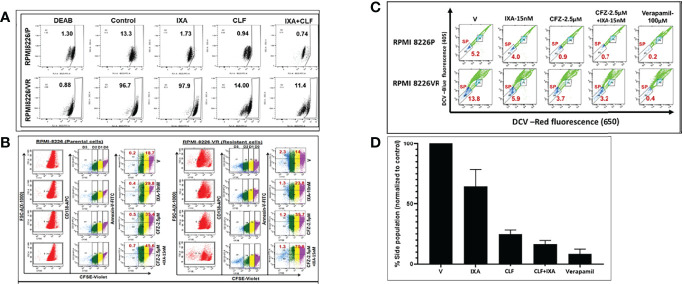
CLF kills myeloma cells with cancer stem-like properties (CSCs). Representative figures showing the RPMI8226 P/VR cell line pair. **(A)** CLF reduces Aldefluor (ALDH) activity: Aldehyde dehydrogenase (ALDH) activity was assessed using the Aldefluor™ kit. The brightly fluorescent ALDH^+^ cells were detected by BD LSR II flowcytometry. i) Innate sensitive and resistant pair; ii) Acquired sensitive and resistant pairs. **(B)** CLF erodes quiescent CD138+ (CFSE bright) cells: Untreated and Treated carboxyfluorescein succinimidyl ester (CFSE; Invitrogen)-stained myeloma cells were labeled with CD138-APC and annexin/V-FITC antibodies and then gated using a BD LSR II flow cytometer into non-dividing cells (CD138+CFSE^bright^) and dividing cell (CD138+CFSE^dim^) population based on CFSE fluorescence intensity. Cells cultured in the presence of colchicine (100ng/ml; Sigma) were used to assess the range of CFSE fluorescence exhibited by cells that were undivided at the end of the culture time. **(C)** CLF erodes Side Population (SP) cells in myeloma: Side population cells in PI-resistant cell lines were investigated using cell-permeable DNA binding Vybrant DyeCycle Violet (DCV; Thermofisher) flow cytometry assays. Positive control sample was incubated with100 μM verapamil (Sigma-Aldrich) for 30 min. **(D)** Summary of Side population results.

**Figures 5A**, **S5A** show baseline ALDH (aldehyde dehydrogenase) activity in PI-resistant HMCLs compared to sensitive HMCLs. While no/very low ALDH activity was observed in the PI-sensitive line FLAM76, we found ALDH activity is remarkably higher (>95%) in clonally derived PI-resistant HMCLs RPMI8226-VR, U266-VR compared to innate resistant LP-1 cell line. CLF caused a significant decrease in ALDH activity, and its effect was comparable with control (**Figure 5A** **)**.

We performed carboxyfluorescein succinimidyl ester (CFSE) assay to evaluate if CLF alone or in combination with PIs could erode quiescent CFSE+ cells in parental (P) and clonally derived PI-resistant (VR) cells, RPMI8226P, RPMI8226VR, U266P, and U266VR. While PIs failed to reduce CFSE-bright (non-dividing) cells, CLF alone or in combination with Ixazomib significantly reduced their number and increased CFSE-dim (dividing cell) population (**Figures 5B**, **S5B**). Remarkably, evaluation of apoptosis in these cells revealed that CLF alone caused apoptosis in both CFSE^bright^ and CFSE^dim^ cells while combining CLF+Ixazomib caused a more robust effect amounting to near-obliteration.

We next gated and selected side population (SP) cells from main populations (MP) using DyeCycle violet, pre- and post- CLF treatment. We found that baseline %SP is higher in resistant cells as compared to parental cells, as shown in (**Figures 5C**, **S5C**). Notably, CLF alone or in combination (CLF+PI) reduced SP in PI- resistant HMCLs (**Figures 5C**, **S5C** **)**. A summary of the results is provided as bar plots in **Figure 5D**.

### Gene Expression Profiling Reveals Potential CLF Mechanism of Action and CLF-Induced Cell Death

Genome-wide transcriptome (next-generation mRNA sequencing or RNAseq) analysis showed 864 genes differed significantly at baseline between the CLF-sensitive and the CLF-resistant groups (p<0.05; fold-difference≠1). Among these, 524 genes had least squares (LS) Mean of 1 or higher in both sensitive and resistant groups. IPA analysis revealed Phagosome Maturation (p=2.45E-05), DNA Methylation and Transcriptional Repression Signaling (p=6.80E-04), Autophagy (p=1.51E-03), BEX2 signaling pathway (p=1.34E-03) as the top canonical pathways associated with CLF sensitivity.

**Figure 6:**
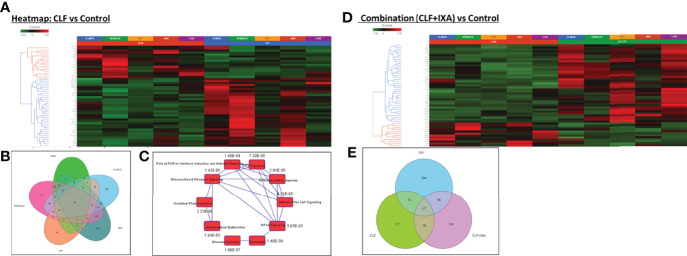
Pre- vs. post-treatment differential gene expression analysis (RNA sequencing). Heatmaps representing top differentially expressed genes following GSA analysis. Gene-specific analysis (GSA) is a statistical modeling method to identify differentially expressed genes. **(A)** CLF single-agent treatment vs. control (untreated); **(B)** Venn Diagram showing 46 significant (p<0.05) genes that were common between all the CLF vs. CON signatures. **(C)** Top canonical pathways predicted by IPA analysis based on the 46-gene shared signature (CLF vs. CON p<0.05). (CLF – Clofazimine; CON – Control/untreated) and **(D)** CLF+Ixazomib combination treatment vs. control (untreated). **(E)** Venn diagram representing common and unique genes between the treatments.

Next, we performed analysis of kinetic changes in gene expression patterns between untreated (baseline) vs. treated (24hrs post-treatment) cells. **Figure S6** shows a heatmap of all the genes with LS mean>=1 that were significant (ANOVA p<0.05) in either CLF vs. CON and/or CLF+IXA vs. CON. Our pair-wise GSA analysis for CLF vs. CON showed that 172 differentially expressed (DE) genes displayed p-value less than 0.05 (|fold-change|>1) in response to single-agent CLF treatment, 24 hours following drug exposure to 5uM CLF. Among these, 45 genes had LS Mean>=1. **Figure S7** shows volcano plots for each pair-wise comparison using gene-specific analysis/GSA. **Figure 6A** shows a heat map of the top DE (CON vs. CLF) genes. When single-agent CLF-induced kinetic changes for each HMCLs were considered separately - 714, 426, 128, 33, and 10 genes were differentially regulated in RPMI8226, FLAM76, JIM3, U266, and LP1, respectively at |fold-change|>2 (p<0.05). This suggests that the number of highly expressed genes decreases with increase in drug resistance, which is consistent with our earlier observation that GEP data may be highly influenced by the DE genes expressed in sensitive HMCLs (22). The Venn diagram in **Figure 6B** shows 46 significant (p<0.05) genes were common between all the CLF vs. CON signatures. IPA analysis (**Figure 6C**) based on these shared gene signatures revealed mitochondrial dysfunction and oxidative phosphorylation as top canonical pathways and predicted downregulation of MYC as the top regulatory gene.

On the other hand, 279 genes changed significantly between untreated vs. CLF+Ixa combination-treated samples (p<0.05; fold-difference≠1). 179 genes had |fold-change|>2 (p<0.05). Among these, 114 genes had LS Mean of 1 or higher in both groups. **Figure 6D** shows a heatmap of the top 50 genes associated with CLF+Ixa combination treatment. Seventy-one genes were common between the two comparisons (CLF vs. Control and CLF+Ixa vs. Control), as shown in the Venn diagram in **Figure 6E**.

**Figure 7:**
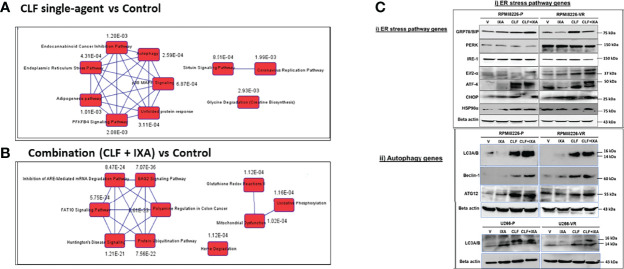
Top pathways potentially associated with CLF mechanism of action and CLF-associated cell death. Ingenuity Pathway analysis: Top 10 canonical pathways predicted by IPA analysis based on the significantly differentially expressed biomarkers. **(A)** CLF single-agent treatment; **(B)** CLF+PI combination treatment. **(C)** Immunoblotting analysis: Pre vs. post-treatment (24hr) western blots showing CLF induces ER stress & Autophagy to kill the myeloma cells.

IPA analysis based on top DE genes revealed Autophagy (p=2.59E-04), Unfolded protein response (p=3.11E-04), and ER stress pathway (p=4.31E-04) as the top canonical pathways associated with CLF treatment gene signatures (**Figure 7A**). BAG2 signaling (p=7.07E-36), FAT10 signaling (p=5.75E-34), polyamine regulation (p=6.61E-33), inhibition of ARE-mediated mRNA degradation pathway (p=8.47E-24) and protein ubiquitination (p=7.56E-22) were among the top canonical pathways for CLF+PI combination treatment (**Figure 7B**). Upstream regulator prediction revealed NR1H4, PPP1R15B, and UCP1 as top upstream regulars for CLF treatment and RICTOR (**Figure S8**) and NFE2L2 for combination treatment. Results from the RNAseq and IPA analysis were confirmed using immunoblotting (**Figure 7C**) as well as CyTOF analysis of ~40 different CLF treatment-induced markers in HMCLs, and PMCs, as shown earlier.

### CLF Shows Superior Anti-Myeloma Cytotoxic Activity Compared to Other PPAR-Gamma Agonists

**Figure 8:**
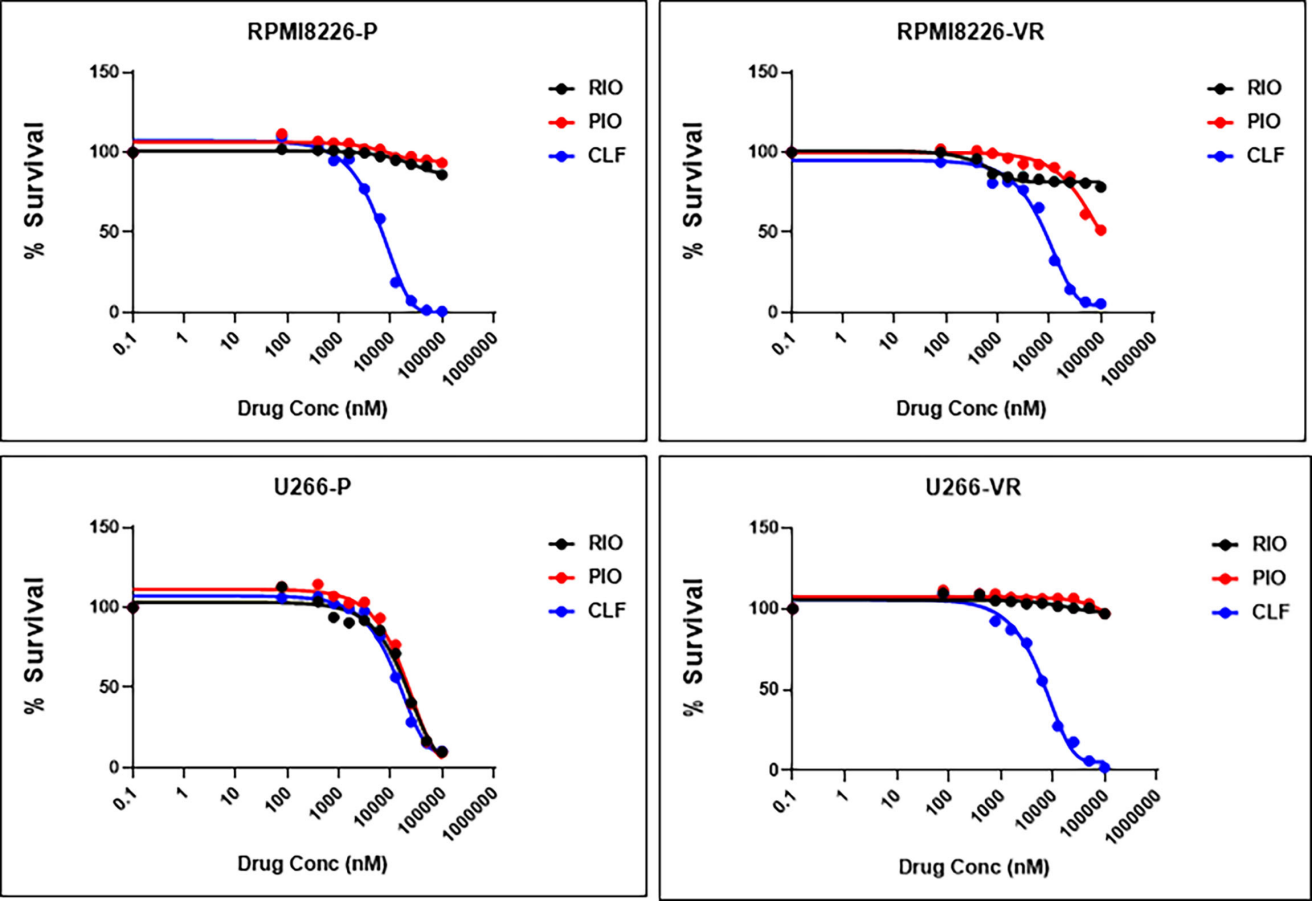
CLF shows superior single-agent cytotoxicity compared to other PPARγ agonists. RIO, Rioglitazone; PIO, Pioglitazone; CLF, Clofazimine.

Next, we compared the anti-myeloma efficacy of CLF with that of other Peroxisome Proliferator Activated Receptor Gamma (PPARg) agonists - rosiglitazone and pioglitazone. Single-agent CLF was found to be the most potent among all the PPARg agonists tested (**Figure 8**).

### CLF Response Is Independent of Aryl Hydrocarbon Receptor (AHR) Expression

An earlier study had reported that the anti-myeloma effect of CLF was due to its inhibition of the AHR-polyamine metabolism axis (12). We evaluated AHR expression using qRT-PCR (TaqMan Real-Time PCR Assays) in 10 myeloma cell lines and compared AHR expression with CLF cytotoxicity. Relative AHR expression (ΔCt) in the myeloma lines was calculated following normalization with Beta-Actin (housekeeping gene). Since the U266 VR cell line showed the lowest relative AHR expression, we used the ΔCt value of this line as the baseline to compute relative fold gene expression of all the HMCLs using the 2^-ΔΔCt^ method (27). The Scatter plot in **Figure S9A** shows that AHR expression was not significantly correlated with CLF response in myeloma cell lines (Spearman r = -0.14; p-value = 0.75). Furthermore, **Figure S9B** shows the cytotoxicity data of the AHR antagonist StemRegenin 1 (SR1) in FLAM76, LP1, U266P, and U266VR cell lines. As represented by IC_50_ values, we found that treatment with SR1 was dependent upon baseline AHR expression (AHR-negative U266 P/VR and FLAM76 lines were highly resistant to SR1 compared to LP1; **Figure S9C**), while CLF treatment response was not correlated with AHR expression (**Figure S9A** **)**.

## Discussion

Drug resistance in multiple myeloma is largely attributed to tumor heterogeneity and inter-individual variations in response to treatment, limiting the therapeutic efficacy in myeloma patients (4, 28, 29). We have earlier demonstrated that wide inter-individual variation exists in response to PI treatment in a panel of HMCLs and PMCs representing the broad spectrum of biological and genetic heterogeneity of myeloma (20). Here, we show significant *in vitro* and *ex vivo* cytotoxicity of CLF against these PI- and IMiD- sensitive and resistant myeloma, both as single agent and in combination with PIs and IMiDs. Further, we performed RNAseq-based next-generation tumor gene expression profiling, single-cell proteomics (CyTOF) analysis, and immunoblotting analysis to identify genes and molecular networks involved in CLF mechanism of action and drug synergy in human myeloma.

Clofazimine is a riminophenazine drug that is approved by the FDA for the treatment of leprosy and has also been shown effective against multidrug-resistant tuberculosis (30). CLF exerts its anti-bacterial actions by producing reactive oxygen species (ROS) like superoxides and hydrogen peroxide (H_2_O_2_) (31). Antioxidants or oxygen scavengers like alpha-tocopherol have been shown to reverse this multidrug-resistance-reversal activity of CLF (32).

Further, CLF displays anti-inflammatory properties resulting in the suppression of immune reactions in leprosy as well as in autoimmune diseases (33). Notably, a very recent study has also shown CLF possesses pan-coronaviral inhibitory activity against the ongoing global COVID-19 (SARS-CoV-2) pandemic (34).

Using *in vitro* screening of 800 drugs, we have earlier identified CLF as a potent inhibitor of cell viability *in vitro* as well as in sensitive and resistant in CML patient samples (10). Although two studies [Bianchi-Smiraglia et al. (12) and Durusu et al. (11)] have shown efficacy of CLF treatment, none of these had deeply explored the mechanisms underlying the synergistic effect of CLF in combination with PI or IMiD therapy or the impact of CLF-based therapy on a large (>10 cell lines) cell line panel or *ex vivo* model systems representing the wide inter- and intra- tumor heterogeneity in myeloma drug response. Thus, we have considerably extended the earlier observations and have explored the CLF mechanism of action using integrated -omics-based methods.

The potential mechanisms behind CLF as an antitumor drug have been previously attributed to its ability to either inhibit the Kv1.3 potassium channel, interfere with the Wnt signaling pathway, or enhance the activity of phospholipase A_2_ (11, 33, 35–37). However, we found *in vitro* CLF cytotoxicity was independent of KV1.3 expression in myeloma cells lines. This was consistent with our observations in CML and also with a recent finding where inhibition of Kv1.3 with PSORA-4 did not change have much effect on CLF cytotoxicity in myeloma cell lines (10, 12, 38).

Previously, we have demonstrated that CLF binds to PPARg, which results in modulation of its transcriptional as well as E3 ubiquitin ligase activity (10). This increased ubiquitin ligase activity of PPARg induces proteasomal degradation of p65, which in turn results in sequential transcriptional downregulation of MYB and PRDX1, resulting in the cellular effects of CLF including regulation of cellular ROS levels (10). This was consistent with our IPA analysis results in myeloma showing BEX2 signaling as the top canonical pathway associated with CLF treatment. The BEX2/NFκB pathway has pro-oncogenic function that includes p65/RelA, NFkB, JUN (39). Earlier studies have shown that BEX2 is the target gene of p65/RelA in cancers and, as a feedback mechanism, regulates the phosphorylation/activity of p65/RelA (40). Further, it was shown that BEX2 functions like an oncogene, activates the NF-kB pathway, and promotes the propagation of human cancer cells (39, 41). In this study, we not only show that IPA analysis predicted downregulation of BEX2/NFκB pathway by Clofazime treatment, our western blotting results show that CLF downregulates NFkB pathway proteins, including p65/RelA, as well as several other proteins belonging to the p65-NFkB-IRF4-Myc axis. Interestingly, a recent study has reported that BEX2 is crucial for maintaining dormant cancer stem cells through the suppression of mitochondrial activity (42).

Further, we also showed that CLF induces ER stress and crosslinks with autophagy for myeloma cell death, in addition to alteration in mitochondrial membrane potential and oxidative phosphorylation. The endoplasmic reticulum (ER) is the primary site for the correct folding and sub-cellular trafficking of proteins, as well as for the initial phase of unfolded protein response (UPR) following ER stress or the accumulation of misfolded or unfolded proteins in the ER (43, 44). The ER stress pathway is involved in i) first, resolving the stress by several mechanisms, including augmenting the removal of unfolded proteins through ER-associated degradation (ERAD) and inducing autophagy (44–46), and ii) in case of failure to resolve the stress, activating cell death or ER stress-induced apoptosis predominantly through the mitochondrial pathway (47).

In our study, using mRNA sequencing, IPA pathway analysis, and western blotting, we showed that CLF treatment induces the proteins involved ER stress pathway, including the ER stress sensors inositol-requiring enzyme 1 (IRE1), PKR-like ER kinase (PERK, EIFA2K3), and activating transcription factor 6 (ATF6), as well as the proteins involved in autophagy in myeloma, which in turn results in induction of apoptosis through the mitochondria-mediated pathway and BCL2-family proteins. This presents a possible mechanism of action of CLF efficacy in myeloma.

Preclinical evidence suggests that synergistic activity of PIs and IMiDs is mediated by enhanced proteasome targeting and the interaction of IMiD and CRBN that inhibits the function of CRBN-associated E3-ubiquitin ligase through direct binding of CRBN to DNA damage-binding protein 1 in a DCX (DDB1-CUL4–X-Box) E3-ubiquitin ligase complex. This initiates a cascade of events leading to NF-κB inhibitory activity and downregulation of IRF4/MYC signaling and MCL1 followed by caspase activation (48–51). This is also consistent with our IPA analysis for CLF+PI combination treatment that showed BAG2 signaling, protein ubiquitination, and FAT10 signaling among the top canonical pathways. FAT10 is a ubiquitin-like protein modifier that serves as a proteasomal degradation signal regulated by ubiquitination (52). Thus, we propose that CLF acts as a PPAR-gamma agonist that synergizes with PIs and IMiDs in myeloma to enhance the downstream cascade of p65-NFkB-IRF4-Myc downregulation followed by ROS-dependent apoptotic cell death. Furthermore, phenotypic and functional characterization by single-cell proteomics (mass cytometry or CyTOF) analysis also showed elevated cleaved caspase-3 levels and downregulation of pS6, IRF4, pCREB, pRB, IKZF1 in HMCLs and patient-derived primary cells, irrespective of PI- or IMiD- resistance. Interestingly, our IPA analysis predicted that gene signatures associated with CLF sensitivity were positively correlated with lenalidomide response genes. Significantly, this might also explain why CLF is effective in the CRBN-deficient IMiD-resistant MM1S LenR cell line. In addition, CyTOF analysis showed less killing of CD3+ T cells by CLF compared to CD19+ B-cells and myeloma cells

Bianchi-Smiraglia et al. (12) postulated that CLF induces cell death in myeloma *via* inhibition of AHR-mediated intracellular polyamine biosynthesis resulting in transcriptional inactivation of the pro-tumorigenic genes ODC1 and AZIN1 (12). Our IPA analysis predicted the AHR-polyamine biosynthesis axis as one of the top canonical pathways based on DE genes associated with CLF+PI combination treatment. However, we observed higher cell kill (low CLF-IC_50_) in the FLAM76 cell line following CLF treatment despite comparatively low AHR gene expression level in this cell line. This points to the possibility that CLF treatment response is either independent of AHR expression or multiple biomolecular networks, including those we have described above, are involved in CLF drug action in myeloma cells. It is to be noted that although our GEP data, IPA analysis, and results from Immunoblotting assays corroborated these findings, further mechanistic validation studies are warranted. Finally, based on the top dysregulated genes following CLF+Ixazomib combination treatment, our Ingenuity Pathway Analysis (IPA) showed downregulation of mTOR signaling as one of the top predictions. This finding underlines the synergistic mechanisms emphasized in the manuscript and lays a framework for further studies in models representing relapsed/refractory myeloma.

A number of studies have shown that the presence of rare subtypes of tumor cell subclones (less-mature cells, or putative cell progenitors or cancer ‘stem cell-like’ cells or CSCs) that are refractory to therapies contribute to drug resistance in various cancers (16, 53–55). Although the exact role and phenotype of these subpopulations in myeloma are unknown, but variously, these cells are thought to include CD138- with memory B‐cells (CD19^+^/CD27^+^), CD38^high^/CD138+ cells, and side population (SP) cells (17, 56–58). Further, myeloma cells with high ALDH activity have also been shown to possess tumorigenesis capacities *in vitro* and *in vivo* (59, 60). Significantly, our study found that CLF is particularly effective against these putative myeloma stem-cell-like subclones with treatment-refractory phenotypes, including quiescent cells/dormant cells, ALDH^+^ cells, and SPs. This demonstrates a unique property of this drug that may be particularly useful in more effective tumor eradication. This is consistent with our previous study where CLF was able to inhibit Leukemic stem cells (LSCs) *via* apoptosis, reduce Aldefluor activity, lower colony number, and induce apoptosis of CD34+-enriched cells in imatinib-resistant CP-CML cells. In addition, we had shown CLF induced significant apoptosis in both committed (CD34+38+) and primitive (CD34+38) patient cells but did not affect normal hematopoietic progenitors in purified CD34+ cells from healthy donors, indicating that CLF targets LSCs (10).

Taken together, we conclude that CLF has strong single-agent cytotoxicity as well as the potential to increase the therapeutic efficacy of standard-of-care drugs (PI and IMiDs) in myeloma, including treatment-resistant and putative stem-like subclones.

Since CLF is FDA-approved, safe (FDA recommended dose is 100mg/day), well-tolerated in patients (61), and is on the WHO’s List of Essential Medicines with low manufacturing cost, re-purposing CLF as a novel clinical trial-ready anti-myeloma agent is an attractive approach for fast and cost-effective drug development.

## Data Availability Statement

The original contributions presented in the study are included in the article/**Supplementary Material**. Further inquiries can be directed to the corresponding author.

## Ethics Statement

The studies involving human participants were reviewed and approved by Mayo Clinic IRB review board. The patients/participants provided their written informed consent to participate in this study.

## Author Contributions

HK designed experimental procedures, conducted cell-based assay experiments, contributed to data analysis and manuscript writing. SM performed RNA sequencing, western blotting, contributed to data analysis and manuscript writing. SC contributed to Western blotting and cell-based assay experiments. NS, ML, and SL performed CyTOF analysis. NM and JP performed direct-to-drug screening analysis on *ex vivo* systems. AKS created the lenalidomide-resistant cell line. MC, SS, LB, SK, and SVR helped with manuscript writing. LBB supervised CyTOF analysis and contributed to manuscript writing. BVN provided logistic support for CyTOF analysis, HMCL procurement and helped with manuscript writing. AKM supervised all project design, performed data analysis, and was responsible for manuscript writing. All authors contributed to the article and approved the submitted version.

## Funding

AKM was supported by a research start-up grant from Auburn University and a grant award from the International Myeloma Foundation. This work was further supported by NCI grant# 1U54CA224018-01 (Sub-Project ID: 8404).

## Conflict of Interest

The authors declare that the research was conducted in the absence of any commercial or financial relationships that could be construed as a potential conflict of interest.

## Publisher’s Note

All claims expressed in this article are solely those of the authors and do not necessarily represent those of their affiliated organizations, or those of the publisher, the editors and the reviewers. Any product that may be evaluated in this article, or claim that may be made by its manufacturer, is not guaranteed or endorsed by the publisher.
