# Supplementary material for: Single-Cell Proteomics and Tumor RNAseq Identify Novel Pathways Associated With Clofazimine Sensitivity in PI- and IMiD- Resistant Myeloma, and Putative Stem-Like Cells
Source: Front Oncol. 2022 May 11;12:842200. doi: 10.3389/fonc.2022.842200 (PMC9130773; doi:10.3389/fonc.2022.842200)
Supplement: Supplementary file 1 [file Presentation_1.pptx]

## Slide 1
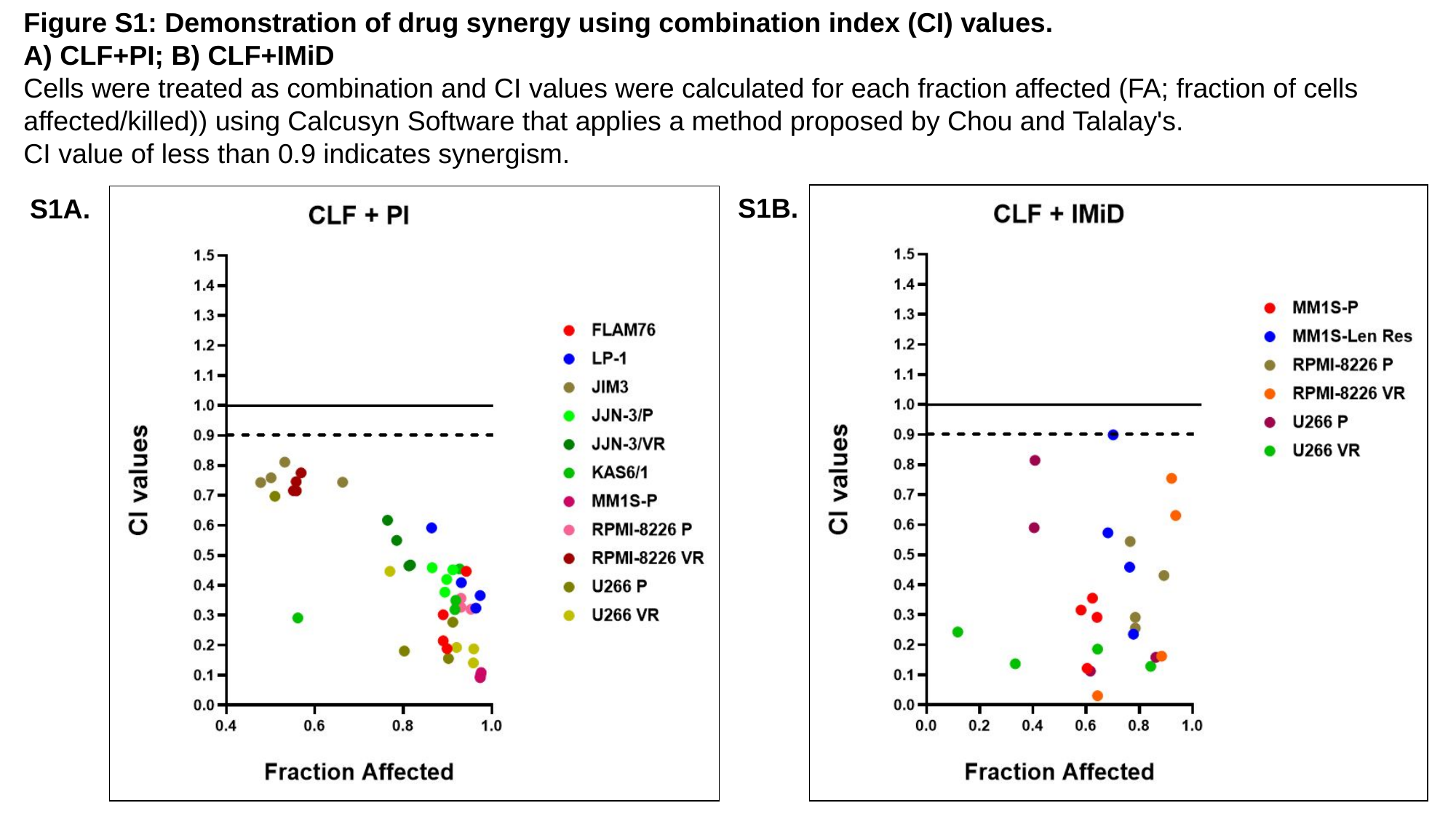

Figure S1: Demonstration of drug synergy using combination index (CI) values.
A) CLF+PI; B) CLF+IMiD
Cells were treated as combination and CI values were calculated for each fraction affected (FA; fraction of cells affected/killed)) using Calcusyn Software that applies a method proposed by Chou and Talalay's.
CI value of less than 0.9 indicates synergism.
S1B.
S1A.

## Slide 2
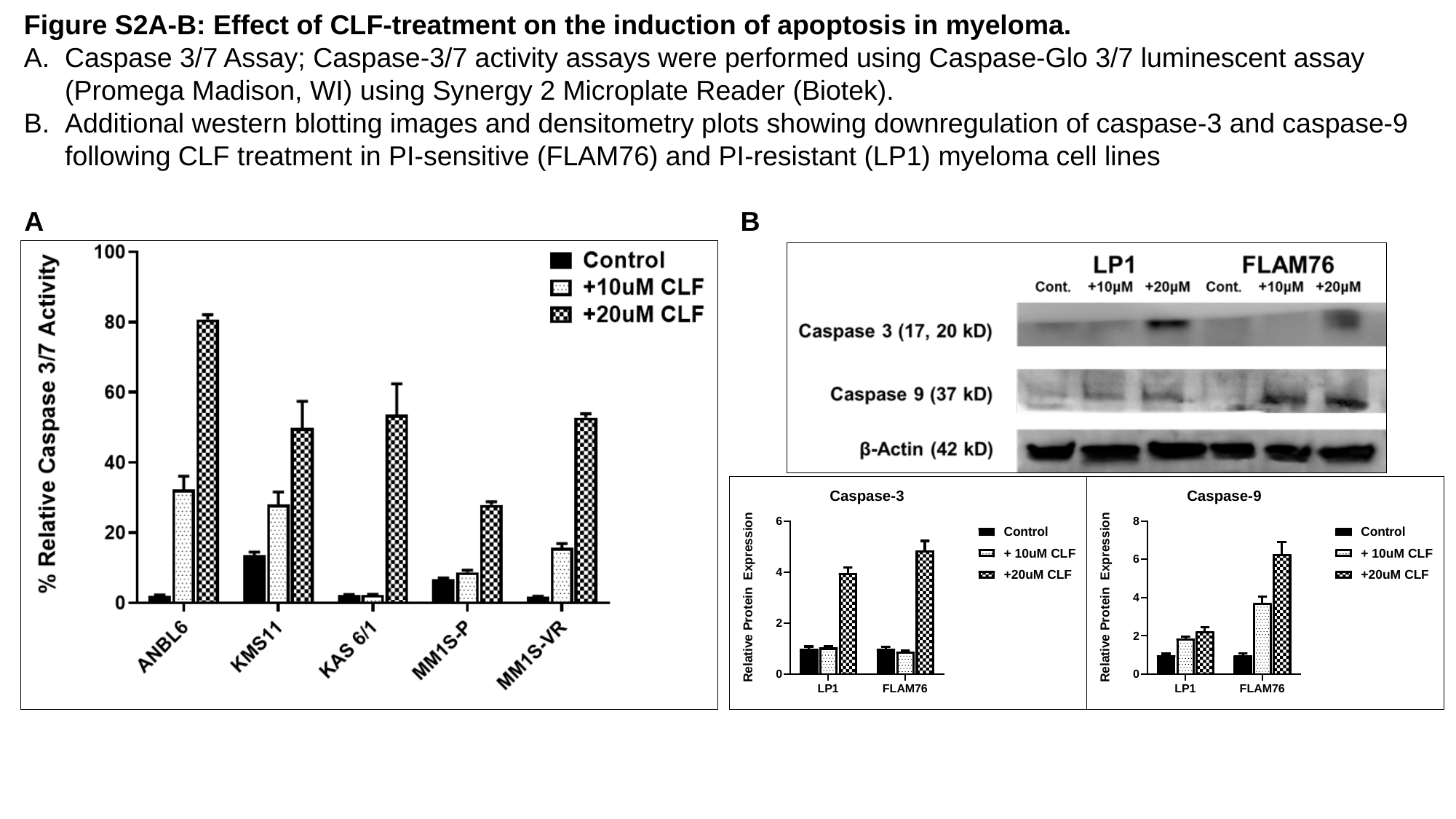

Figure S2A-B: Effect of CLF-treatment on the induction of apoptosis in myeloma.
Caspase 3/7 Assay; Caspase-3/7 activity assays were performed using Caspase-Glo 3/7 luminescent assay (Promega Madison, WI) using Synergy 2 Microplate Reader (Biotek).
Additional western blotting images and densitometry plots showing downregulation of caspase-3 and caspase-9 following CLF treatment in PI-sensitive (FLAM76) and PI-resistant (LP1) myeloma cell lines
A
B

## Slide 3
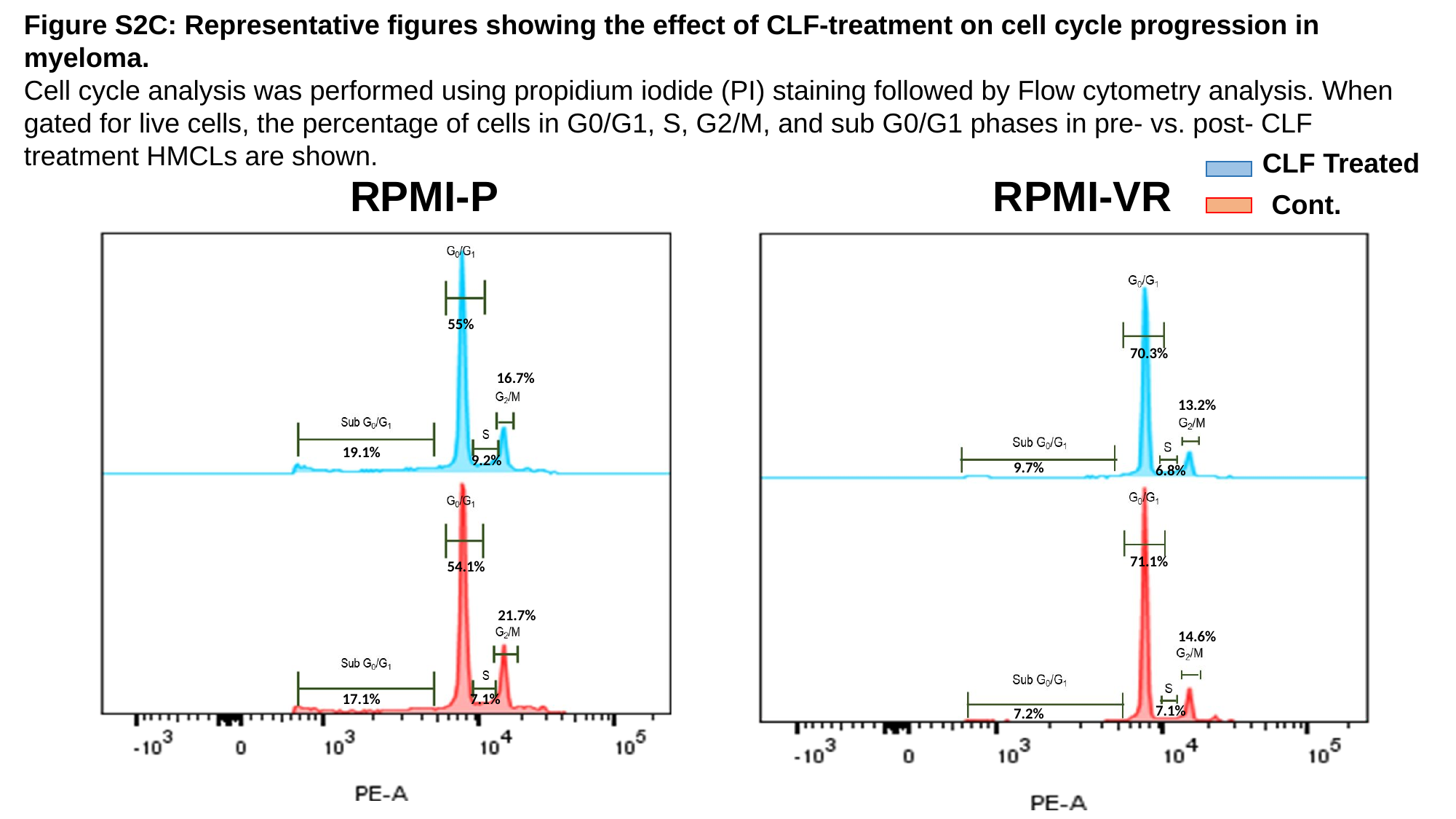

Figure S2C: Representative figures showing the effect of CLF-treatment on cell cycle progression in myeloma.
Cell cycle analysis was performed using propidium iodide (PI) staining followed by Flow cytometry analysis. When gated for live cells, the percentage of cells in G0/G1, S, G2/M, and sub G0/G1 phases in pre- vs. post- CLF treatment HMCLs are shown.
CLF Treated
RPMI-P RPMI-VR
Cont.
55%
70.3%
16.7%
13.2%
19.1%
9.2%
9.7%
6.8%
71.1%
54.1%
21.7%
14.6%
7.1%
17.1%
7.1%
7.2%

## Slide 4
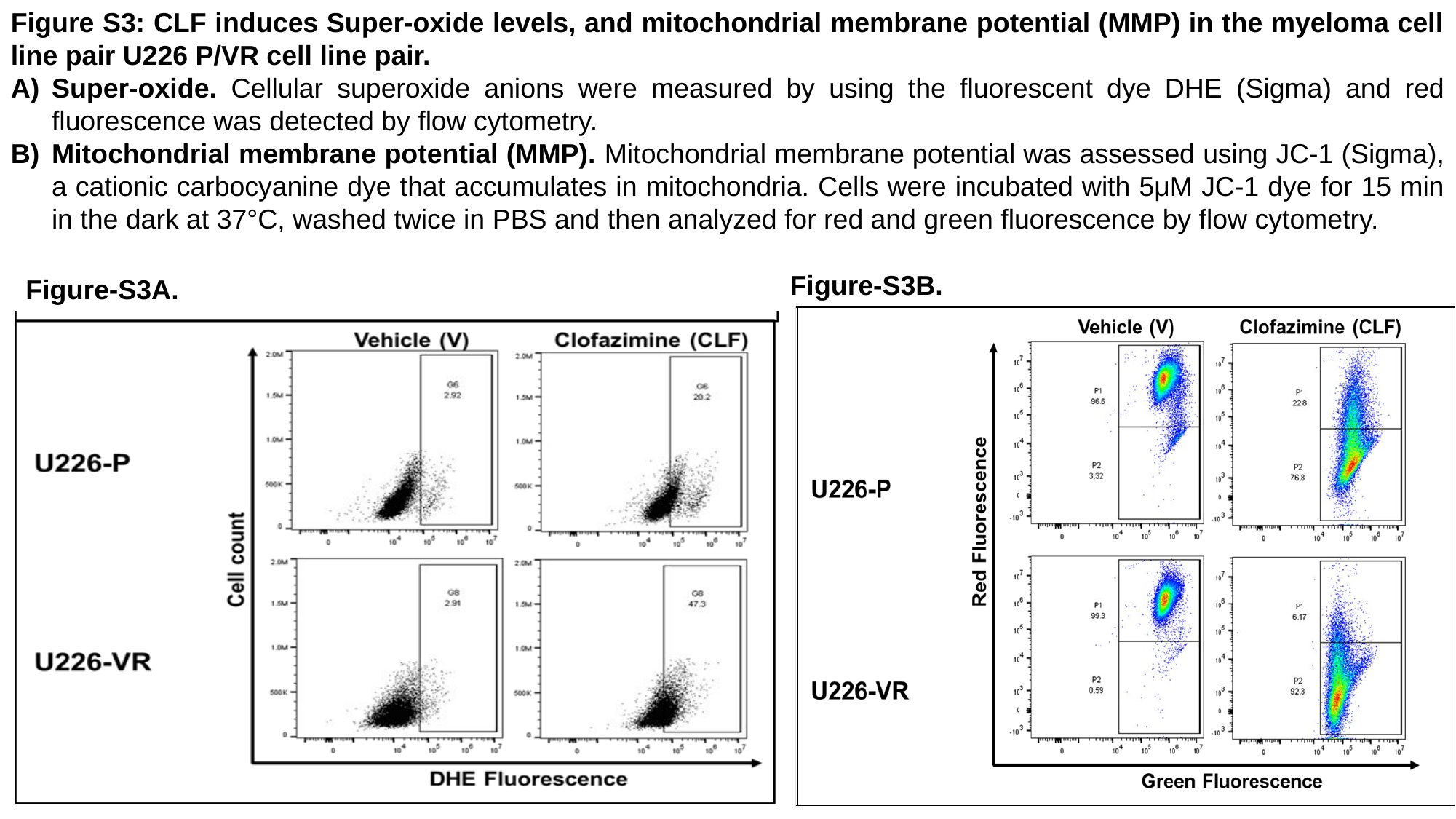

Figure S3: CLF induces Super-oxide levels, and mitochondrial membrane potential (MMP) in the myeloma cell line pair U226 P/VR cell line pair.
Super-oxide. Cellular superoxide anions were measured by using the fluorescent dye DHE (Sigma) and red fluorescence was detected by flow cytometry.
Mitochondrial membrane potential (MMP). Mitochondrial membrane potential was assessed using JC-1 (Sigma), a cationic carbocyanine dye that accumulates in mitochondria. Cells were incubated with 5μM JC-1 dye for 15 min in the dark at 37°C, washed twice in PBS and then analyzed for red and green fluorescence by flow cytometry.
Figure-S3B.
Figure-S3A.

## Slide 5
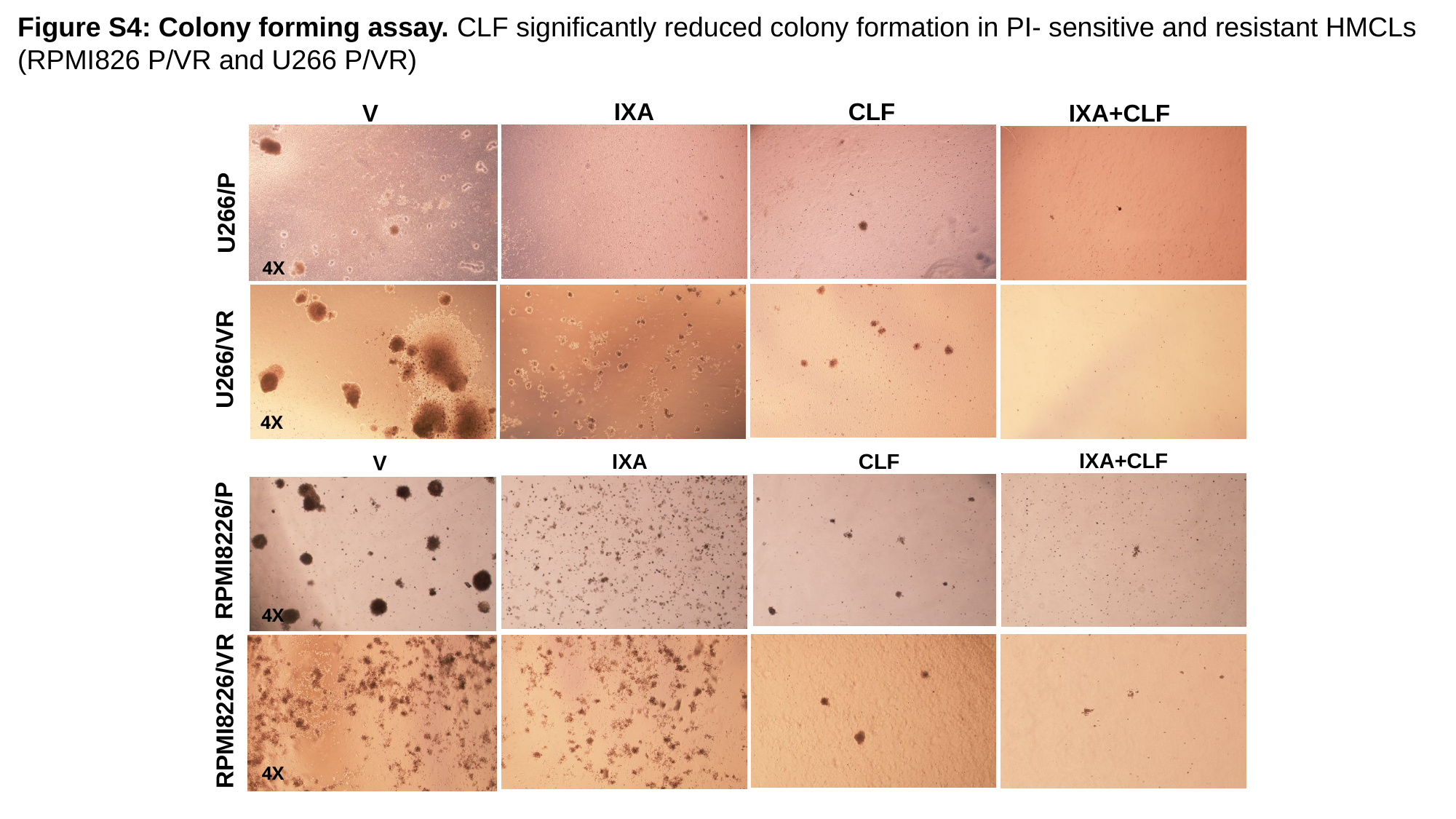

Figure S4: Colony forming assay. CLF significantly reduced colony formation in PI- sensitive and resistant HMCLs (RPMI826 P/VR and U266 P/VR)
IXA
CLF
IXA+CLF
V
U266/P
4X
U266/VR
4X
IXA+CLF
IXA
CLF
V
RPMI8226/P
4X
RPMI8226/VR
4X

## Slide 6
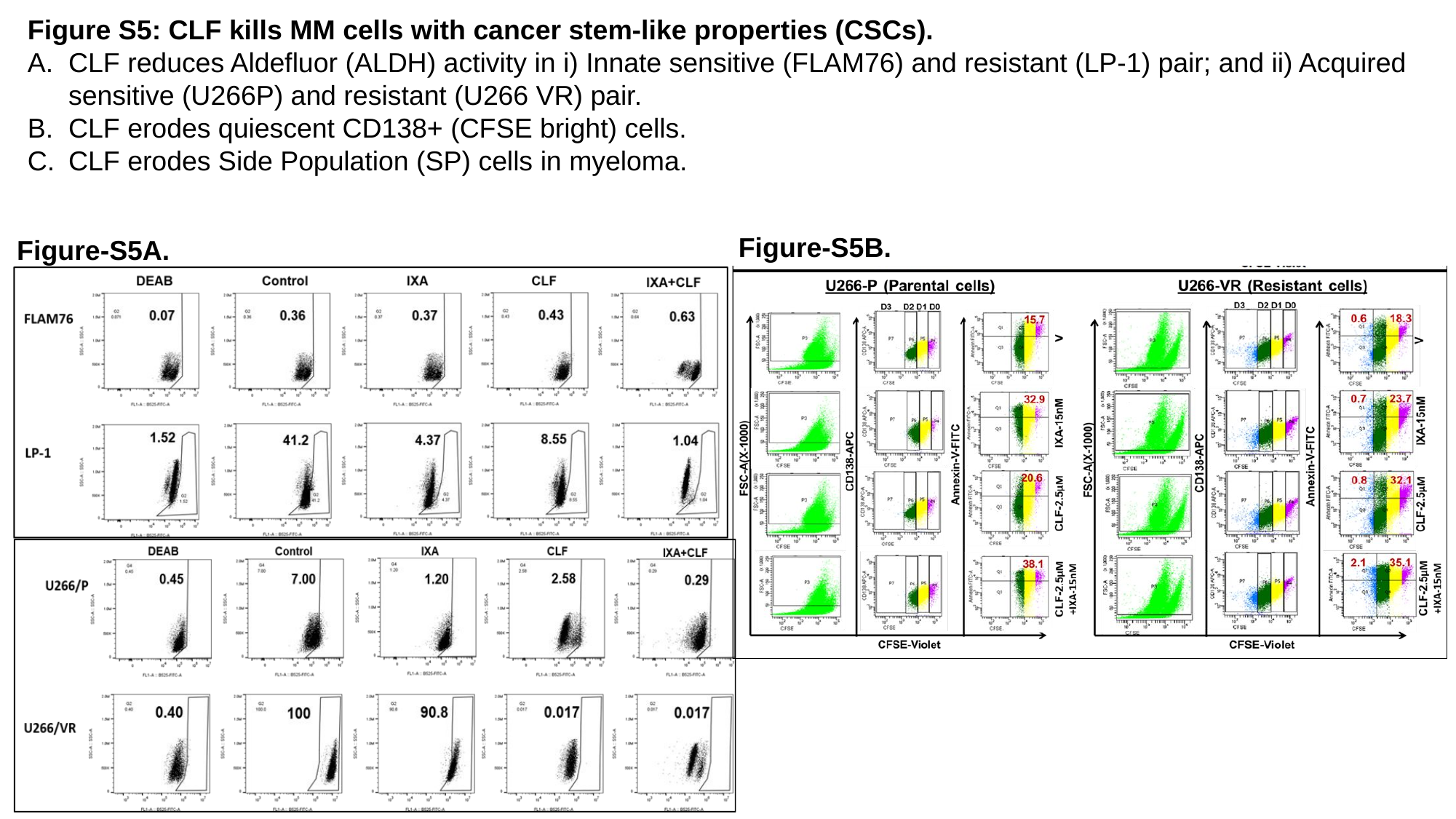

Figure S5: CLF kills MM cells with cancer stem-like properties (CSCs).
CLF reduces Aldefluor (ALDH) activity in i) Innate sensitive (FLAM76) and resistant (LP-1) pair; and ii) Acquired sensitive (U266P) and resistant (U266 VR) pair.
CLF erodes quiescent CD138+ (CFSE bright) cells.
CLF erodes Side Population (SP) cells in myeloma.
Figure-S5A.
Figure-S5B.

## Slide 7
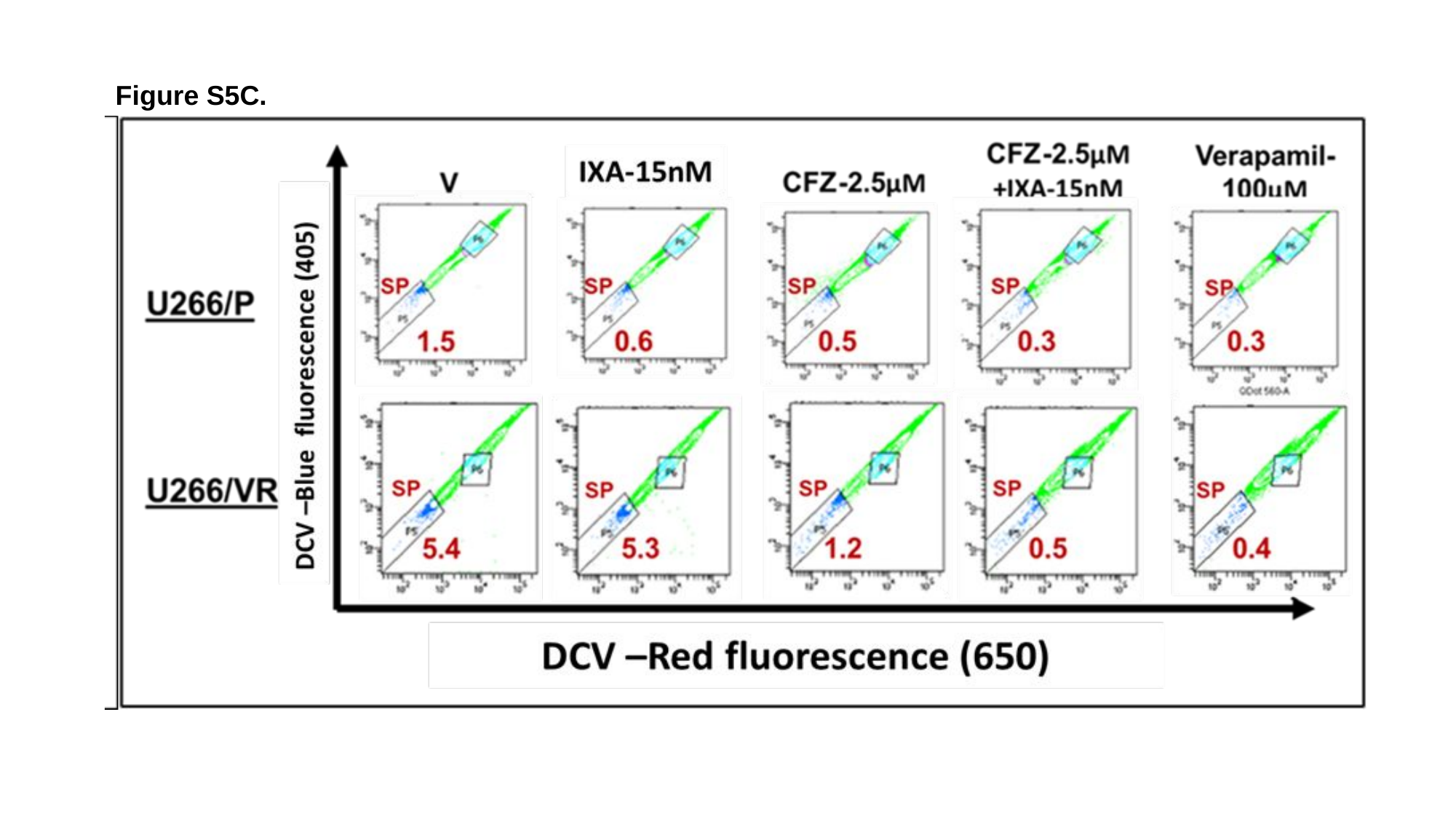

Figure S5C.

## Slide 8
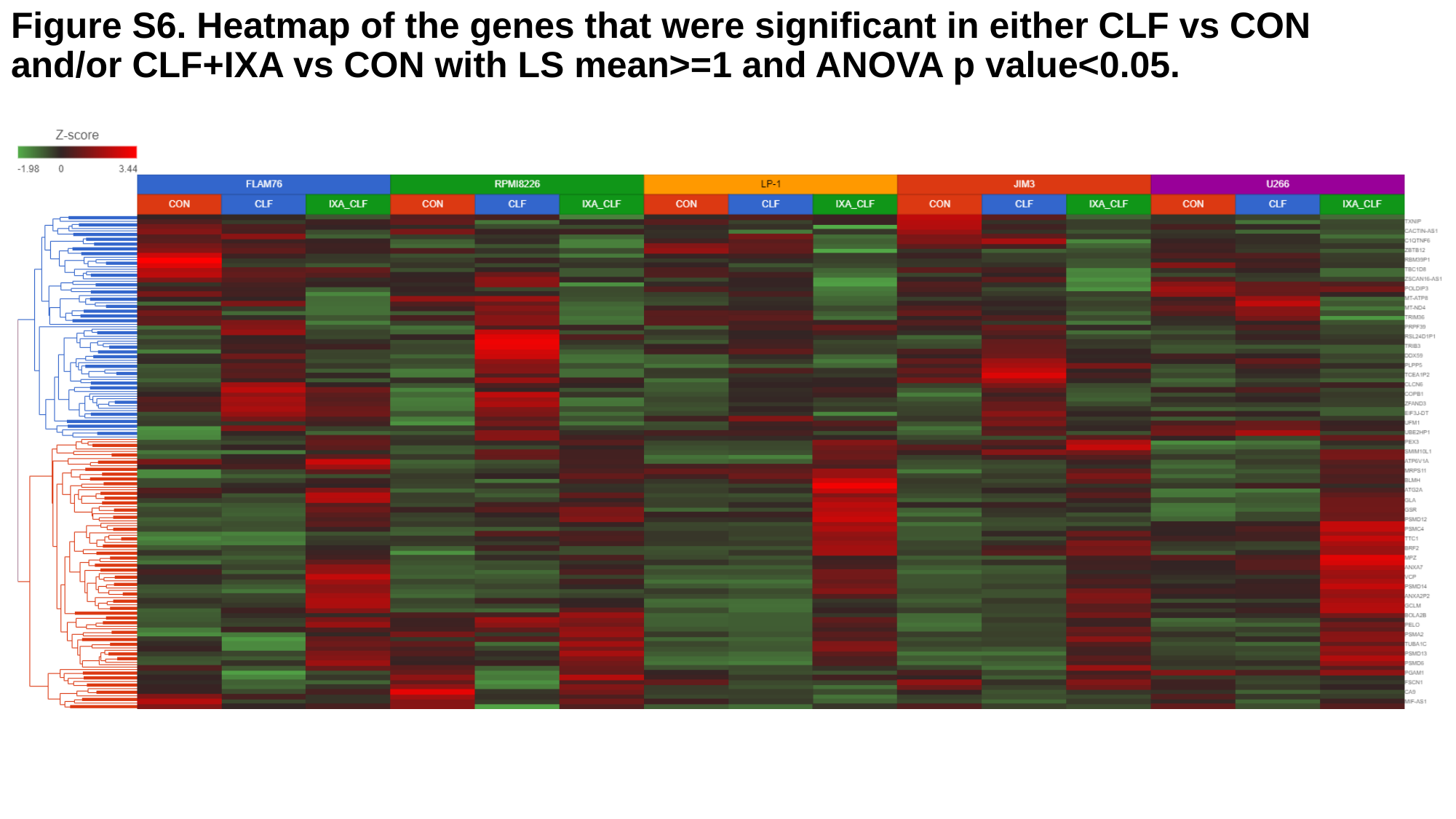

# Figure S6. Heatmap of the genes that were significant in either CLF vs CON and/or CLF+IXA vs CON with LS mean>=1 and ANOVA p value<0.05.

## Slide 9
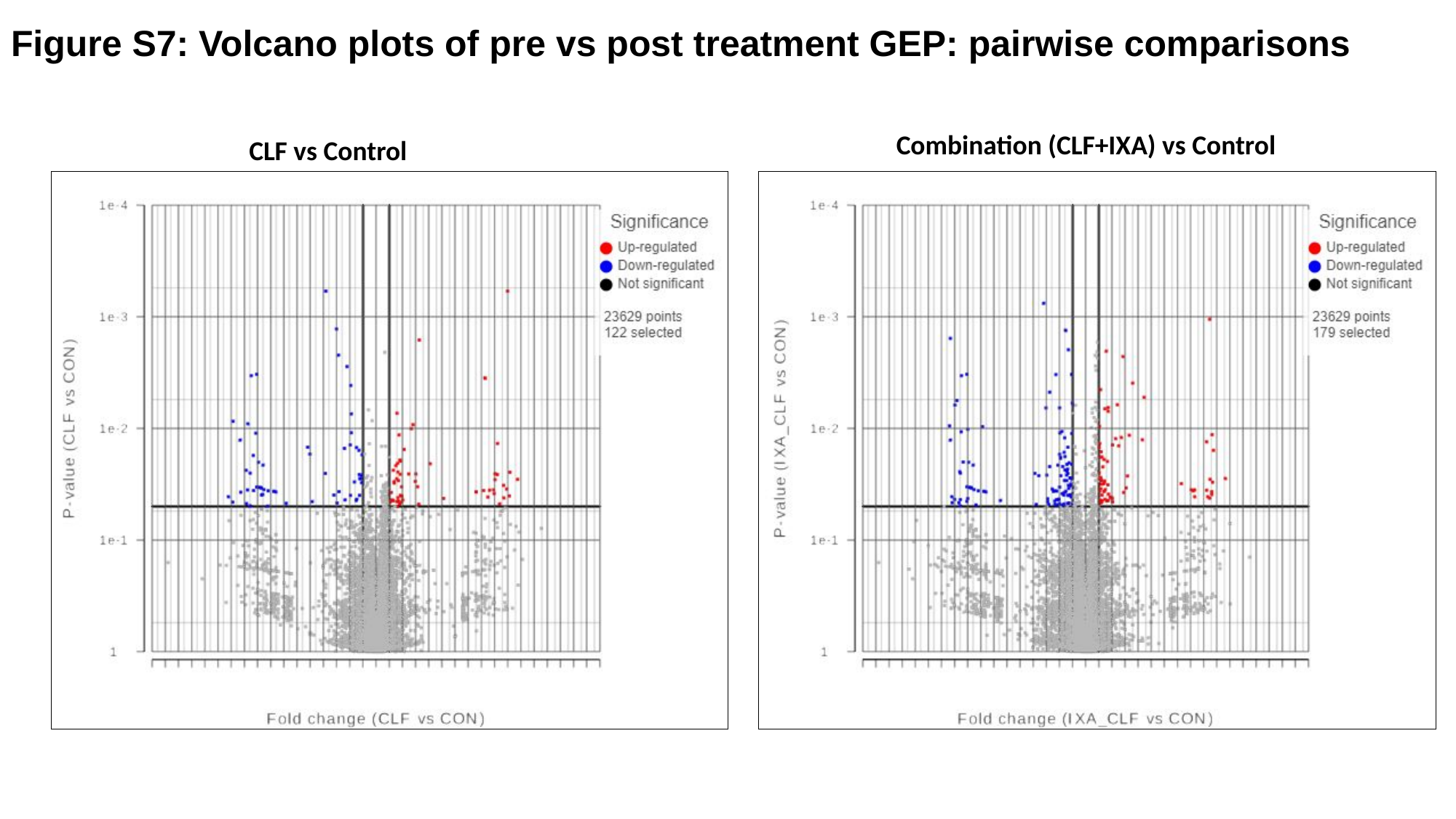

# Figure S7: Volcano plots of pre vs post treatment GEP: pairwise comparisons
Combination (CLF+IXA) vs Control
CLF vs Control

## Slide 10
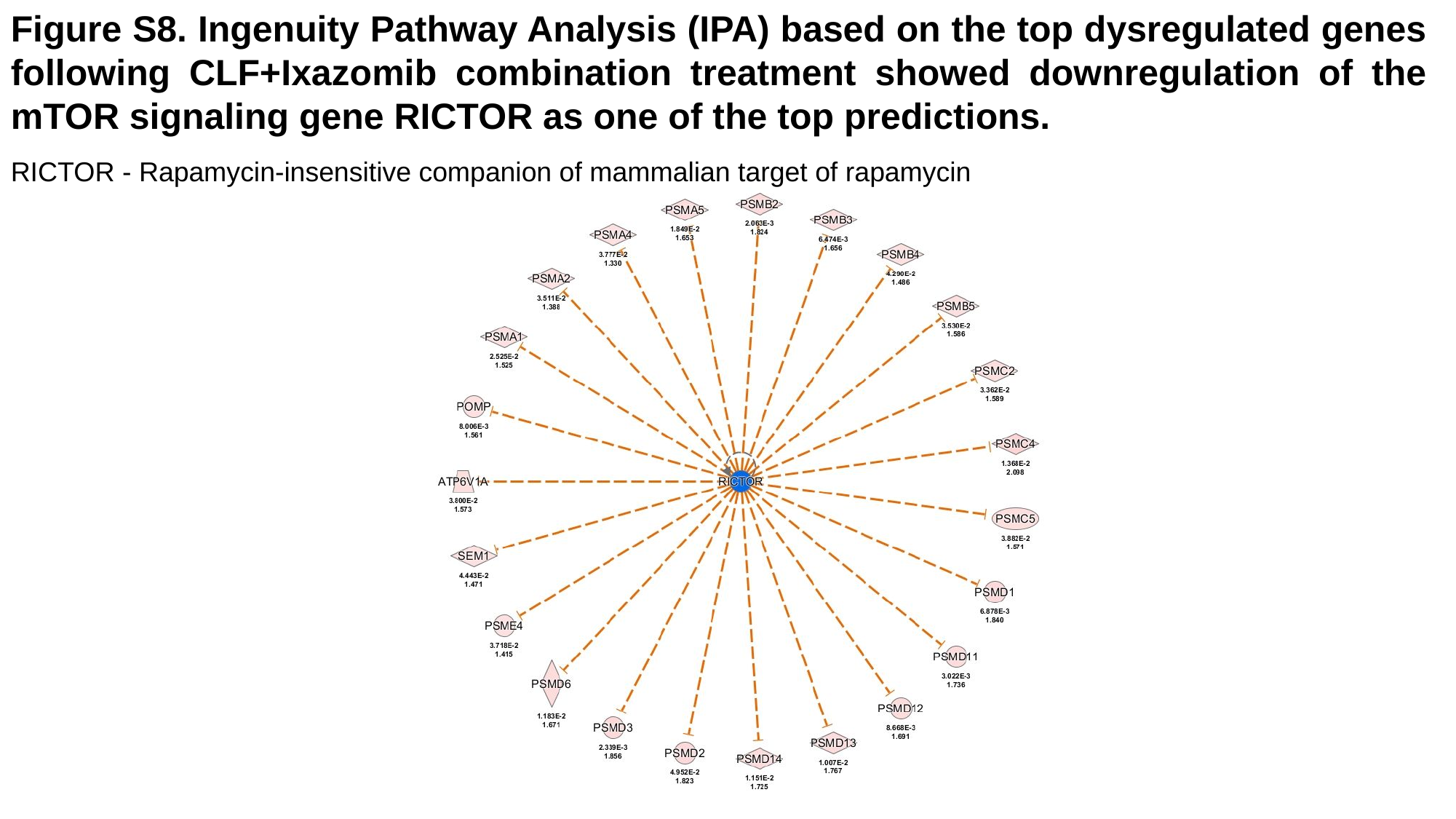

Figure S8. Ingenuity Pathway Analysis (IPA) based on the top dysregulated genes following CLF+Ixazomib combination treatment showed downregulation of the mTOR signaling gene RICTOR as one of the top predictions.
RICTOR - Rapamycin-insensitive companion of mammalian target of rapamycin

## Slide 11
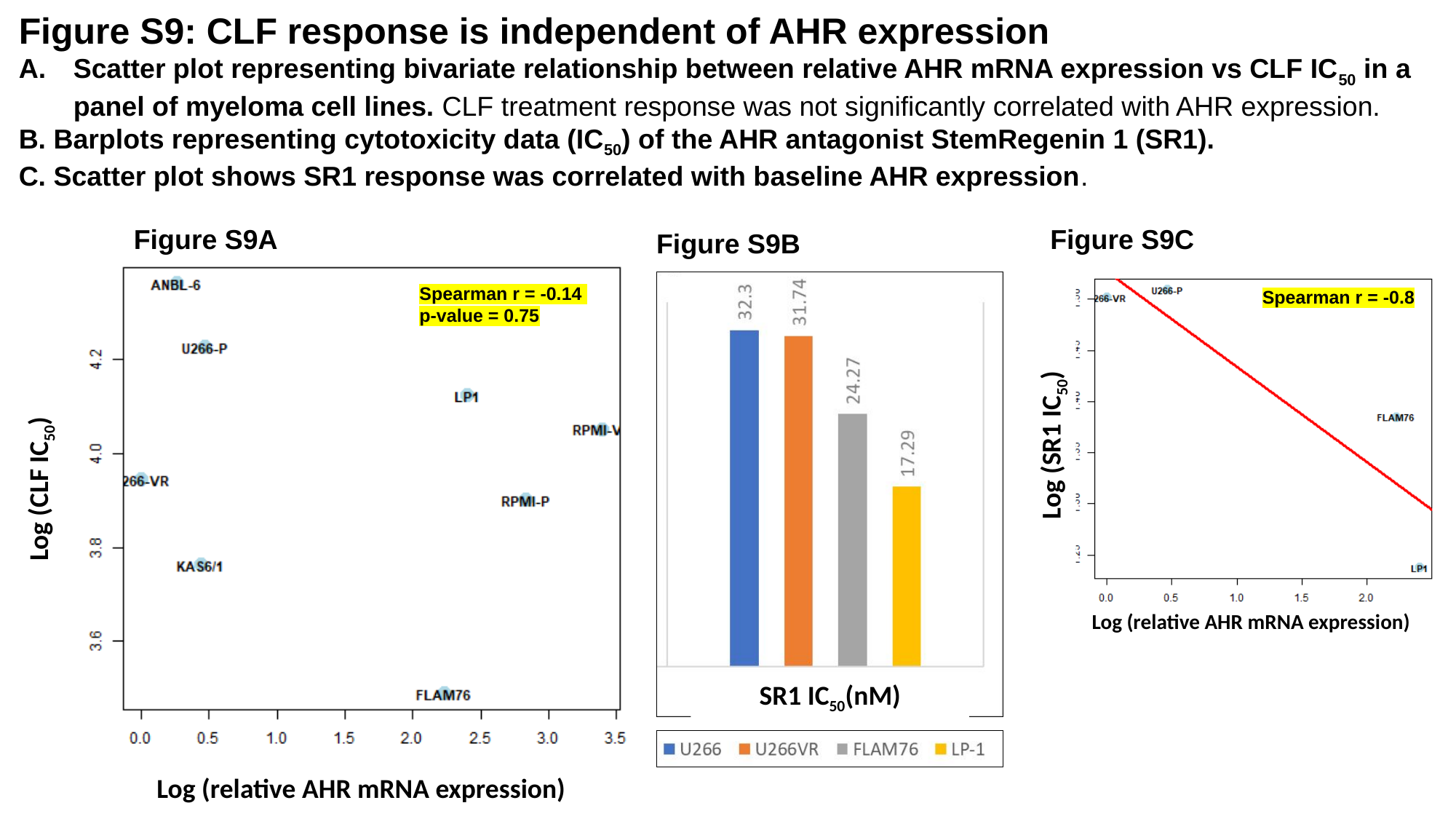

Figure S9: CLF response is independent of AHR expression
Scatter plot representing bivariate relationship between relative AHR mRNA expression vs CLF IC50 in a panel of myeloma cell lines. CLF treatment response was not significantly correlated with AHR expression.
B. Barplots representing cytotoxicity data (IC50) of the AHR antagonist StemRegenin 1 (SR1).
C. Scatter plot shows SR1 response was correlated with baseline AHR expression.
Figure S9A
Figure S9C
Figure S9B
Spearman r = -0.14
p-value = 0.75
Spearman r = -0.8
Log (SR1 IC50)
Log (CLF IC50)
Log (relative AHR mRNA expression)
SR1 IC50(nM)
Log (relative AHR mRNA expression)

## Slide 12
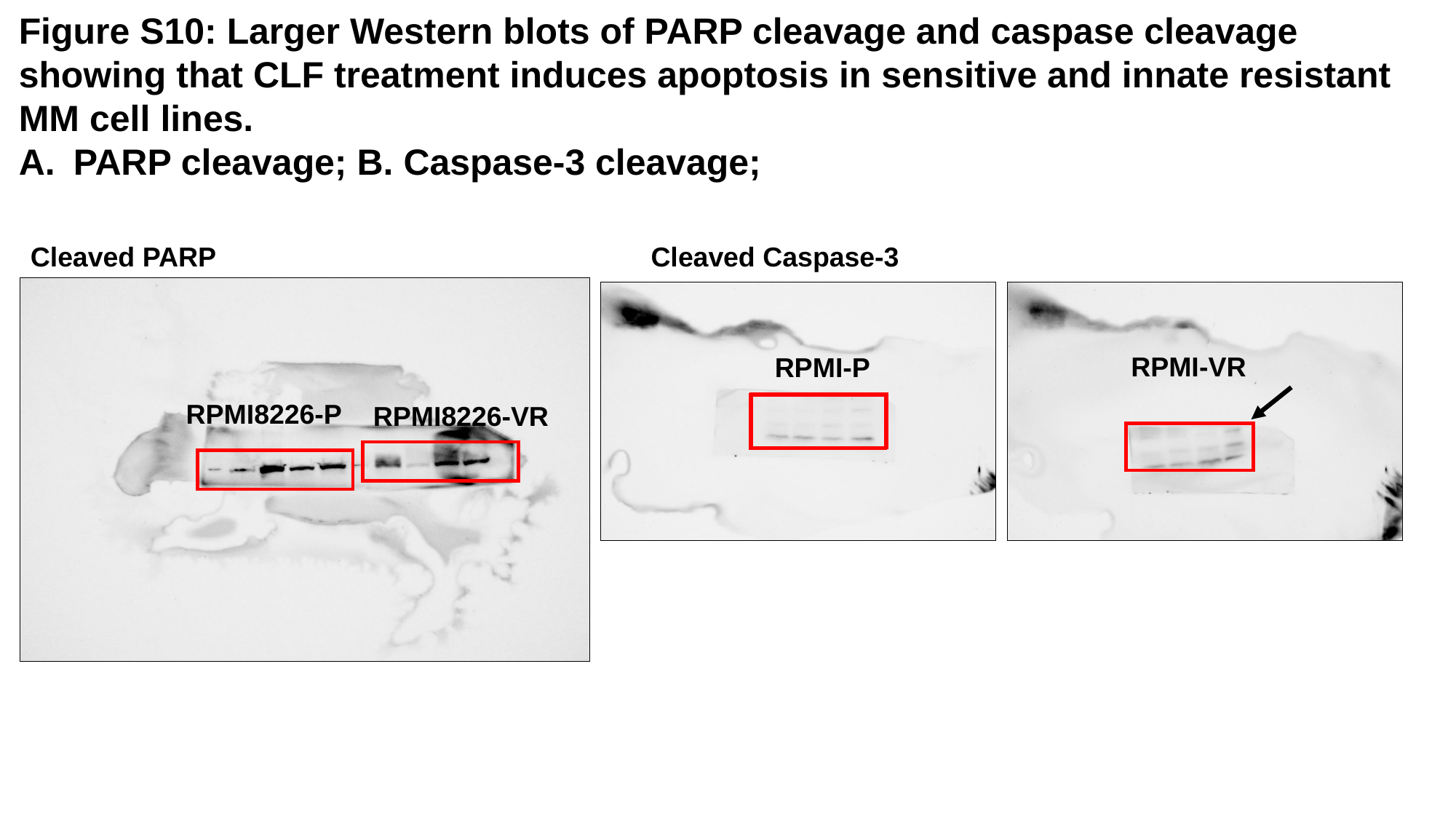

Figure S10: Larger Western blots of PARP cleavage and caspase cleavage showing that CLF treatment induces apoptosis in sensitive and innate resistant MM cell lines.
PARP cleavage; B. Caspase-3 cleavage;
Cleaved Caspase-3
RPMI-VR
RPMI-P
Cleaved PARP
RPMI8226-P
RPMI8226-VR
