# Supplementary material for: Single-Cell Proteomics and Tumor RNAseq Identify Novel Pathways Associated With Clofazimine Sensitivity in PI- and IMiD- Resistant Myeloma, and Putative Stem-Like Cells
Source: Front Oncol. 2022 May 11;12:842200. doi: 10.3389/fonc.2022.842200 (PMC9130773; doi:10.3389/fonc.2022.842200)
Supplement: Supplementary file 2 [file Table_1.docx]

**Table S1. List of drugs, reagents, antibodies, and kits used in the study.** All the drugs were dissolved in dimethyl sulfoxide (DMSO) and stored at -20ºC.

|  | **Antibody/Kits** | **Manufacturer** |
| --- | --- | --- |
| **Drugs** |  |  |
|  | Clofazimine (CLF) | Sigma-Aldrich (St Louis, MO) |
|  | (±)-Verapamil hydrochloride | Sigma-Aldrich (St Louis, MO) |
|  | Ixazomib (Ixa) | Takeda Pharmaceuticals Inc., Deerfield, IL, USA |
|  | Bortezomib (Btz) | Takeda Pharmaceuticals Inc., Deerfield, IL, USA |
|  | Carfilzomib (Cfz) | Amgen |
|  | Oprozomib (Opz) | Amgen |
|  | Lenalidomide | Selleck Chemicals |
| **Reagents/Kits** |  |  |
|  | MethoCult™ H4434 Classic / Methylcellulose-based medium with recombinant cytokines | Stem cell Technologies |
|  | FITC Annexin V Apoptosis Detection Kit | BD bioscience |
|  | Vybrant™ DyeCycle™ Violet Stain | Thermo Fisher Scientific |
|  | CellTrace™ CFSE Cell Proliferation Kit | Thermo Fisher Scientific |
|  | Aldefluor™ Kit | Stem cell Technologies |
|  | Caspase-Glo® 3/7 Assay System | Promega |
|  | CellTiter-Glo 2.0 Assay | Promega |
|  | ′,7′-Dichlorofluorescein diacetate | Sigma-Aldrich (St Louis, MO) |
| **Antibodies** |  |  |
|  | GRP-78 | Cell signaling Technology (CST) |
|  | phospho-PERK (Thr980) (16F8) | Cell signaling Technology (CST) |
|  | Total-PERK | Cell signaling Technology (CST) |
|  | Phospho-eIF2α (Ser51) | Cell signaling Technology (CST) |
|  | Total-eif2α | Cell signaling Technology (CST) |
|  | CHOP | Cell signaling Technology (CST) |
|  | IRE-1 | Cell signaling Technology (CST) |
|  | ATF-4 | Cell signaling Technology (CST) |
|  | ATF-6 | Cell signaling Technology (CST) |
|  | NRF2 (D1Z9C) XP(R) | Cell signaling Technology (CST) |
|  | Cleaved caspase-3 | Cell signaling Technology (CST) |
|  | Cleaved caspase-8 | Cell signaling Technology (CST) |
|  | Cleaved caspase-9 | Cell signaling Technology (CST) |
|  | Cleaved PARP | Cell signaling Technology (CST) |
|  | Bax | Cell signaling Technology (CST) |
|  | BCL2 | Cell signaling Technology (CST) |
|  | Survivin | Cell signaling Technology (CST) |
|  | MCL-1 | Cell signaling Technology (CST) |
|  | LCIIA/B | Cell signaling Technology (CST) |
|  | Beclin-1 | Cell signaling Technology (CST) |
|  | Atg12 | Cell signaling Technology (CST) |
|  | HSP70 | Cell signaling Technology (CST) |
|  | HSP90 | Cell signaling Technology (CST) |
|  | β-catenin | Cell signaling Technology (CST) |
|  | c-Myc | Cell signaling Technology (CST) |
|  | p65 | Cell signaling Technology (CST) |
|  | IRF4 | Cell signaling Technology (CST) |
|  | PRDX1 | Cell signaling Technology (CST) |
|  | CyclinD1 | Cell signaling Technology (CST) |
|  | Monoclonal Anti-β-Actin−Peroxidase (Mouse) | Sigma-Aldrich (St Louis, MO) |
|  | Goat anti-Mouse/Rabbit IgG (H+L) Secondary antibody (HRP conjugated) | Thermo Fisher Scientific |
|  | APC anti-mouse CD138 (Syndecan-1) | Bio-legend |
|  | APC Rat IgG2a, κ Isotype Ctrl ab | Bio-legend |
| **CyTOF reagents** |  |  |
|  | Benzonase | Millipore Sigma |
|  | Cell-ID Cisplatin | Fluidigm |
|  | Maxpar Fix I Buffer | Fluidigm |
|  | Veri-Cells™ PBMC | BioLegend |
|  | Maxpar Cell Staining Buffer | Fluidigm |
|  | Formaldehyde solution | Thermo Scientific |
|  | Cell-ID™ Intercalator-Ir | Fluidigm |
|  | Maxpar Fix and Perm Buffer | Fluidigm |
|  | Maxpar Cell Acquisition Solution | Fluidigm |
|  | Cell-ID 20-Plex Pd Barcoding Kit | Fluidigm |

**Supplementary Table S2**. Cell surface and intracellular targets for CyTOF analysis

| SN. | 1. **Cell surface targets** | **Metal Tag** | **Source/Manufacturer** | **Catalog No.** |
| --- | --- | --- | --- | --- |
| 1. | CD45 | 89Y | Fluidigm | 3089003B |
| 2. | CD38 | 114Nd | Fluidigm | 3144014B |
| 3. | CD138 | 168Er | Fluidigm | 3168009B |
| 4. | CD3 | 141Pr | Fluidigm | 3141019B |
| 5. | CD56 | 149Sm | Fluidigm | 3149021B |
| 6. | CD19 | 169Tm | Fluidigm | 3169011B |
| 7. | CD81 | 145Nd | Fluidigm | 3145007B |
| 8. | CD20 | 147Sm | Fluidigm | 3147001B |
| 9. | CD34 | 148Nd | Fluidigm | 3148001B |
| 10. | CD274 | 159Tb | Fluidigm | 3159029B |
| 11. | CD27 | 167Er | Fluidigm | 3167006B |
| 12. | CD229 | 174Yb | Fluidigm | 3174017B |
| 13. | CD16 | 209Bi | Fluidigm | 3209002B |
| 14. | CD86 | 150Nd | Fluidigm | 3150020B |
| 15. | CD117* | 173Yb | BioLegend | 313223 |
| 16. | CD28* | 154Sm | BioLegend | 302937 |
| 17. | CD147* | 161Dy | BioLegend | 306206 |
| 18. | CD71* | 170Er | BioLegend | 334102 |
| **SN.** | 1. **Intracellular targets** | **Metal Tag** | **Source/Manufacturer** | **Catalog No.** |
| 1. | 1kBα | 164Dy | Fluidigm | 3164004A |
| 2. | pERK 1/2 [T202/Y204] | 171Yb | Fluidigm | 3171010A |
| 3. | pStat3 [Y705] | 158Gd | Fluidigm | 3158005A |
| 4. | IRF4 | 155Gd | Fluidigm | 3155014B |
| 5. | IKZF1 | 143Nd | Fluidigm | 3143024B |
| 6. | Ki-67 | 172Yb | Fluidigm | 3172024B |
| 7. | pS6 [S235/S236] | 175Lu | Fluidigm | 3175009A |
| 8. | MCL 1 | 163Dy | Fluidigm | 3163006A |
| 9. | Caspase 3/Cleaved | 142Nd | Fluidigm | 3142004A |
| 10. | pAkt [S473] | 152Sm | Fluidigm | 3152005A |
| 11. | p38 [T180/Y182] | 156Gd | Fluidigm | 3156002A |
| 12. | pRb [S807/811] | 166Er | Fluidigm | 3166011A |
| 13. | pCREB [S133] | 165Ho | Fluidigm | 3165009A |
| 14. | IKZF3 | 162Dy | Fluidigm | 3162032B |
| 15. | c-Myc | 176Yb | Fluidigm | 3176012B |
| 16. | Ig kappa/light chain | 160Gd | Fluidigm | 3160005B |
| 17. | Ig lambda/light chain | 151Eu | Fluidigm | 3151004B |
| 18. | BCL-2* | 153Eu | BioLegend | 658702 |
| 19. | Cyclin D1* | 146Nd | Santa Cruz Biotechnology | SC-8396 |

*In-house conjugated antibodies using the X8 polymer MaxPAR antibody conjugation kit (Fluidigm) as per manufacturer’s instructions.
